# Polymorph selectivity of an AIE luminogen under nano-confinement to visualize polymer microstructures†

**DOI:** 10.1039/c9sc04239c

**Published:** 2019-11-22

**Authors:** Michidmaa Khorloo, Yanhua Cheng, Haoke Zhang, Ming Chen, Herman H. Y. Sung, Ian D. Williams, Jacky W. Y. Lam, Ben Zhong Tang

**Affiliations:** Department of Chemistry, The Hong Kong Branch of Chinese National Engineering Research Center for Tissue Restoration and Reconstruction, Institute for Advanced Study and Development of Chemical and Biological Engineering, The Hong Kong University of Science and Technology Clear Water Bay Kowloon Hong Kong China tangbenz@ust.hk; State Key Laboratory for Modification of Chemical Fibers and Polymer Materials, College of Materials Science and Engineering, Donghua University Shanghai 201620 China cyh@dhu.edu.cn; HKUST-Shenzhen Research Institute No. 9 Yuexing 1st RD, South Area, Hi-tech Park, Nanshan Shenzhen 518057 China; Center for Aggregation-Induced Emission, SCUT-HKUST Joint Research Institute, State Key Laboratory of Luminescent Materials and Devices, South China University of Technology Guangzhou China

## Abstract

Despite the huge progress of luminescent molecular assemblies over the past decade, it is still challenging to understand their confined behavior in semi-crystalline polymers for constrained space recognition. Here, we report a polymorphic luminogen with aggregation-induced emission (AIE), capable of selective growth in polymer amorphous and crystalline phases with distinct color. The polymorphic behaviors of the AIE luminogen embedded within the polymer network are dependent on the size of nano-confinement: a thermodynamically stable polymorph of the AIE luminogen with green emission is stabilized in the amorphous phase, while a metastable polymorph with yellow emission is confined in the crystalline phase. The information on polymer crystalline and amorphous phases is transformed into distinct fluorescence colors, allowing a single AIE luminogen as a fluorescent marker for visualization of polymer microstructures in terms of amorphous and crystalline phase distribution, quantitative polymer crystallinity measurement, and spatial morphological arrangement. Our findings demonstrate that confinement of the AIE luminogen in the polymer network can achieve free space recognition and also provide a correlation between microscopic morphologies and macroscopic optical signals. We envision that our strategy will inspire the development of other materials with spatial confinement to incorporate AIE luminogens for various applications.

## Abstract 2

A polymorphic AIEgen is capable of selective growth in amorphous and crystalline polymer phases with distinct color for microstructure visualization.

## Graphical abstract

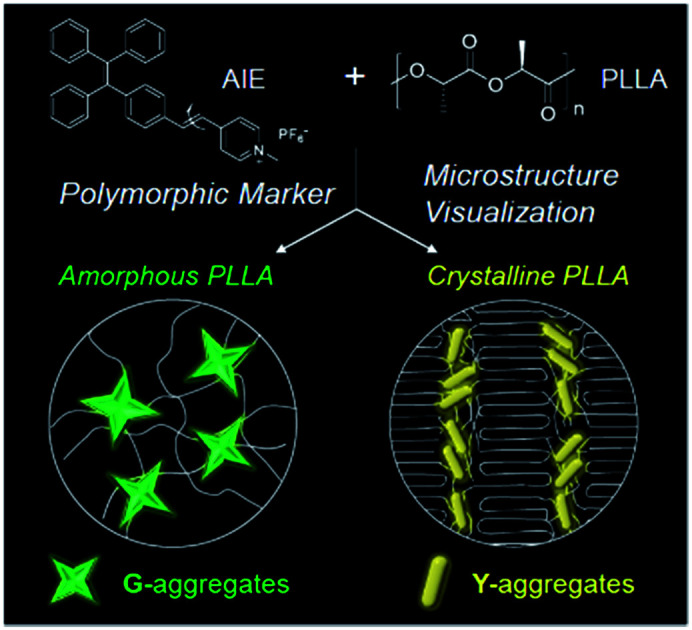

## Introduction

Polymorphism is a widespread phenomenon in nature and industrial materials. It describes the ability of a substance to exist in more than one form or crystal structure that have different arrangements of molecules but with the same chemical formula.^1^ Polymorphism is of great importance to determine the functional properties of materials.^2^ For example, in natural minerals, polymorphs of graphite and diamond exhibit distinct mechanical properties; in pharmaceuticals, the same medicine with different polymorphs shows a great impact on dissolution rates and bioactivity.^3^ In the field of opto-electronics, small π-conjugated molecules act as rigid building blocks to build ordered assembled structures, which have potential application in data storage and advanced sensing.^4,5^ The molecular structure, together with packing mode, conformations and their intermolecular noncovalent interaction, collectively determines the molecular assembled structure.^6–8^ The assembled structure has been demonstrated to directly influence the photophysical properties of the π-conjugated molecules, such as photoluminescence color, quantum yield and emission life.^9^

Various polymorphic crystals of a single π-conjugated molecule may exist, with each polymorph exhibiting different emissions owing to differences in its molecular assembled structure.^6^ Most luminescent π-conjugated systems show two emissive states, but multicolour luminogens have also been reported by using metastable states.^6,10–12^ Experiments to control different polymorphs are normally carried out in bulk solution and driven by the self-organization process.^13,14^ However, uncertainty and inhomogeneity are usually resulted, hampering the precise control over the polymorphism of the molecular systems across large areas.^13^ Recent studies show that spatial confinement can be used to address the above problem.^15,16^ Owing to the large ratios of surface area to volume of confined nano-space, the embedded crystals show a series of phase behaviours, such as polymorph selective formation, size-dependent polymorphism, and thermodynamic stabilization of metastable phases.^15^ Although the nano-pores of porous glass powders^17^ and polymer monoliths^18^ have been utilized as spatial confinements, reliable materials that can retain continuous active layers when deformed are still required to suit for future flexible devices.^19^ The fact that the polymer network is generally composed of intrinsically free volumes at the nanoscale that originated from the gaps between entangled polymer chains^20^ encourages research in polymers. Importantly, previous diffusion measurements have suggested that the size of free volume varies in crystalline and amorphous regions of semi-crystalline polymers.^21^ These studies show the potential of achieving polymorphism control of molecular systems by using semi-crystalline polymers as tailorable nano-constrained environments.

In this context, a polymorphic molecular system that is sensitive to polymer microenvironments should be a prior choice. A molecular system characterized with aggregation-induced emission (AIE) is well suited for such purposes. AIE describes a general phenomenon in which molecules are non-luminescent in solution but give intense emission when aggregated.^22–24^ The restriction of intramolecular motion (RIM) has been recognized as the mechanism of the AIE phenomenon, which prevents the dissipation of excited-state energy through nonradiative decay channels.^25–27^ According to the RIM mechanism, the AIE systems have been successfully exploited to examine local microenvironments.^28–33^ Moreover, the twisted 3D molecular conformation and weak intermolecular interactions of AIE molecules make them show multiple structural transformability, generating variable polymorphs with different emission properties.^12,34–37^ Taking advantage of the AIE features and the tailorable confined space of semi-crystalline polymers, polymorph selectivity in macroscopic and continuous samples can be anticipated.

**Fig. 1:**
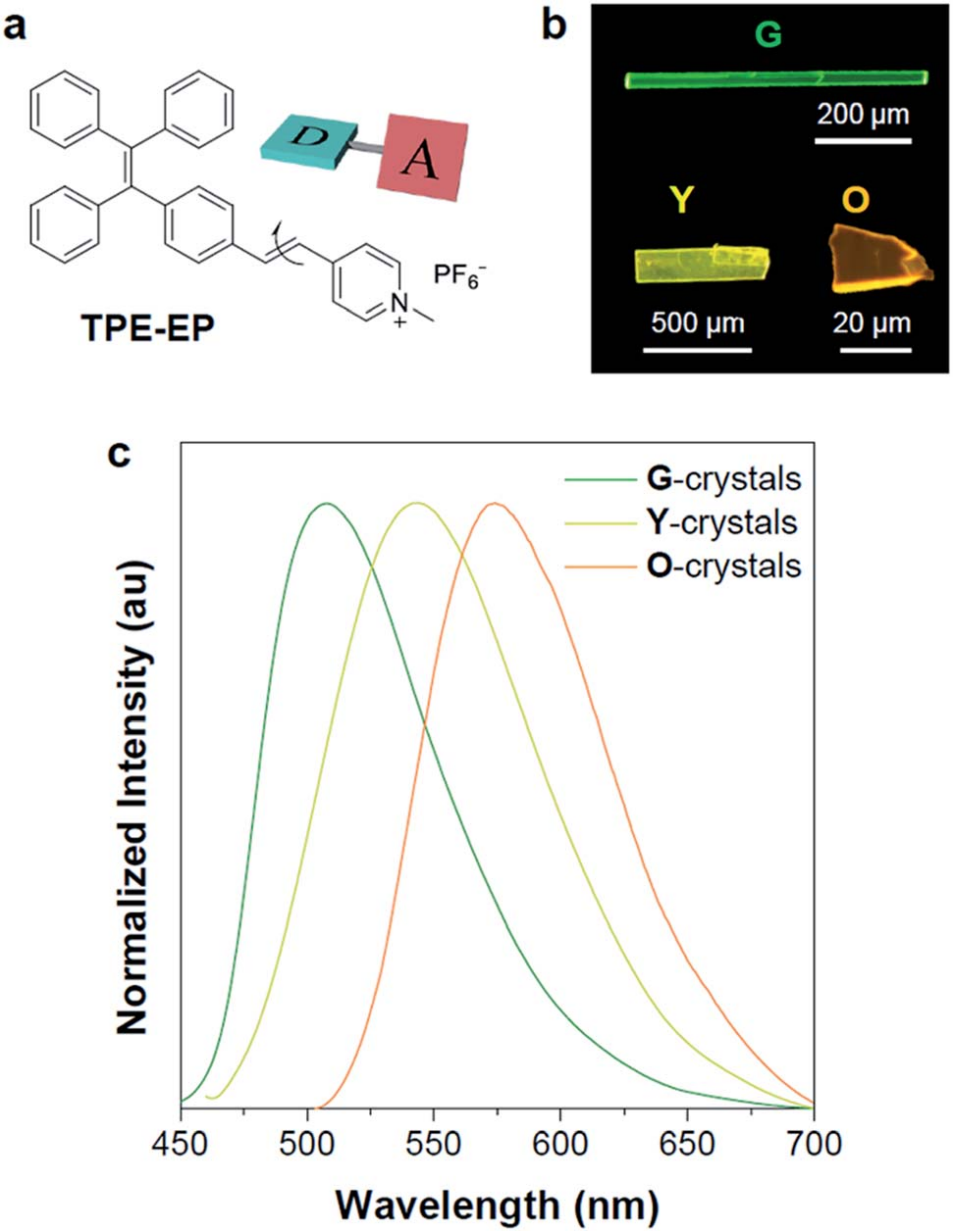
Structural and optical properties of TPE-EP. (a) D–A based AIE luminogen. (b) Fluorescence photographs of polymorphic G-, Y-, and O-crystals as well as their (c) corresponding emission spectra. Excitation wavelength: 405 nm.

In an effort to demonstrate the above hypothesis, herein, we report a D–A structured, polymorphism-dependent emissive AIE luminogen, which is crystallized within the semi-crystalline polymer of poly(l-lactide) (PLLA) as confined spaces and exhibits polymorph selectivity behaviour. An amphiphilic D–A based AIE molecule (denoted as TPE-EP) was used by attaching a pyridinium salt unit to the tetraphenylethene (TPE) group *via* a double bond (Fig. 1a), yielding segregated solid structures by separating its hydrophilic and hydrophobic units. The fluorescence of TPE-EP in the aggregate state depends on the polymorphic forms: crystalline forms G (thermodynamically stable state), Y (metastable state) and O (metastable state), which exhibit green, yellow and orange emission, respectively. The amphiphilic structure forces the molecules to form segregated nanocrystals in hydrophobic PLLA: in amorphous PLLA, molecular aggregates in polymorph G are stabilized in a loose network; in crystalline PLLA, molecular aggregates in polymorph Y are confined in between lamellae. Such a phenomenon allows TPE-EP to operate as a fluorescent marker for visualization of polymer microstructures, including amorphous and crystalline phase distribution, quantitative polymer crystallinity measurement, and spatial morphological arrangement. The AIE molecules with suitable polymorphic characteristics are expected to have broad applications in diverse polymers, facilitating both mechanistic studies of polymorphism control in nano-confinement and the development of fluorescent materials with tunable emission.

## Results and discussion

### Molecular information and polymorphic properties

In this study, a highly twisted AIE luminogen with a D–A structure named TPE-EP was crystallized within the semi-crystalline PLLA network. TPE-EP consists of three subunits: a hydrophobic TPE as the electron donor unit, a hydrophilic pyridinium salt group as the electron acceptor unit, and a double bond as the spacer unit (Fig. 1a). Initially, polymorphs of TPE-EP obtained from bulk solution were studied in order to understand the relationship between photophysical behaviour and structural arrangement. By controlling precipitation conditions, three crystalline forms (G, Y and O) can be produced. Fig. 1b shows the photos of G-, Y-, and O-crystals taken under a fluorescence microscope. Green photoluminescence was observed for G-crystals under UV excitation (405 nm) with an emission maximum at 507 nm, quantum yield (*Φ*_F_) of 0.21 and life time (*τ*) of 2.1 ns (Table S1, ESI;†Fig. 1c, green solid line). The crystal structure of G was determined by single-crystal X-ray diffraction (XRD). G-crystals were found to be monoclinic (*P*2_1_/*n*, *a* = 11.2525(2) Å, *b* = 9.21940(13) Å, *c* = 59.1397(9) Å, *α* = 90°, *β* = 93.8053(14)°, *γ* = 90° at 100.03 K, *Z* = 4, goodness of fit (GOF) = 1.036, calculated density: 1.384 g cm^−3^, Table S2, ESI†). The polymorphic Y-crystals (Fig. 1b) were produced by slow evaporation of a tetrahydrofuran/hexane mixture of TPE-EP and showed a yellow emission (*λ*_em_ = 543 nm) under UV irradiation (405 nm, Fig. 1c, yellow solid line). Y-crystals were found to exhibit a *Φ*_F_ of 0.32 (Table S1, ESI†). The crystal structure of Y was also monoclinic with a space group of *P*2_1_/*c* (*a* = 24.8939(7) Å, *b* = 9.1783(2) Å, *c* = 12.5794(3) Å, *α* = 90°, *β* = 98.168(3)°, *γ* = 90° at 100.01 K, *Z* = 4, goodness of fit (GOF) = 1.019, calculated density: 1.390 g cm^−3^, Table S2, ESI†). O-crystals showed orange emission (*λ*_em_ = 575 nm) but it is too brittle to be studied by single crystal XRD. A thermal phase transformation of Y into G was revealed at an elevated temperature of 116 °C (Fig. S1, ESI†). No reverse phase was observed when the sample was cooled. Y-crystals were only obtained by a re-dissolution and recrystallization process. A similar phenomenon was also observed in O-crystals (Fig. S1, ESI†). The thermal analyses suggest that G is a thermodynamically favourable form, while Y- and O-crystals are metastable (Fig. S2, ESI†).^38^

**Fig. 2:**
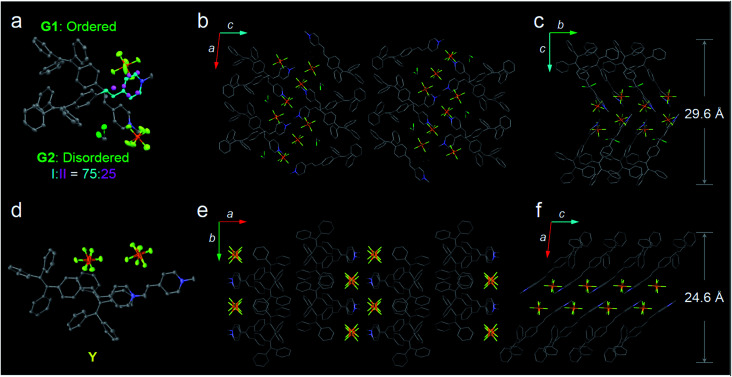
Crystal structures of polymorphs G and Y. (a) Ellipsoid drawing (50% probability level) of the crystal structure of G adopting the cross-packing mode. A dimer unit consisting of the crystallographically ordered unit (G1) and disordered unit (G2) with two conformations. Conformers I and II are colored with blue and pink, respectively. Occupancies of conformers I and II are 75% and 25%, respectively. (b) Crystal structure of G depicted as a stick model viewed along the *b* axis. (c) Crystal structure of G viewed along the *a* axis. (d) Ellipsoid drawing of the crystal structure of Y adopting the parallel-stacking mode. (e and f) Crystal structures of Y. The hydrogen atoms in G- and Y-crystals are omitted for clarity.

The formulae of the G-crystals and Y-crystals are C_69_H_58_Cl_2_F_12_N_2_P_2_ (TPE-EP dimer with dichloromethane at a ratio of 1 : 2) and C_34_H_28_F_6_NP, respectively (Fig. 2a–f and Table S2, ESI†). In the case of G-crystals, G1 and G2 form a pair in which two molecules are cross packed with two TPE units arranged on the same sides (Fig. 2a). No apparent overlap of their π-planes was found (Fig. S3, ESI†). In addition, an orientational disorder (G2) was observed in the crystal structure because of the adoption of two crystallographically independent conformations with occupancies of 75 : 25 (I : II). The ordered (G1) and disordered (G2) conformers are alternately placed from row to row (Fig. S4, ESI†). Besides, infinite channels exist in G-crystals, which are enough to accommodate small solvent molecules in the crystalline lattice (Fig. 2b).^39^ The presence of voids in G-crystals can explain the observed larger crystal volume (6121.68 Å^3^/8 molecules) relative to that of Y-crystals (2845.01 Å^3^/4 molecules) (Table S2, ESI†). The inclusion of dichloromethane solvent in G-crystals results in an increase of molecular flexibility and decrease of radiative transition.^34^ These cross-stacking dimers account for the needle-like crystals (Fig. 1b),^40^ which are stabilized by multiple intermolecular interactions (Fig. S5, ESI†).

Compared with G-crystals, the increased overlapping between the pyridine rings and the double bonds of Y-crystals accounts for the stronger intermolecular interactions to induce a red-shift in emission (Fig. 2d and S3, ESI†). The two adjacent molecules adopt comparatively parallel head-to-head arrangement, in which one molecule slides to its neighbour along the molecular long axis. They show a strong 2D growth trend along the crystallographic *a*–*b* plane (Fig. 2e) which results in the formation of plate-like crystals (Fig. 1b).^41^ Meanwhile, more and stronger interatomic hydrogen bonds (C–H⋯F: 2.041–2.555 Å, Fig. S6, ESI†) are observed in Y-crystals than in G-crystals (C–H⋯F: 2.413–2.666 Å, Fig. S5, ESI†), which further rigidifies the molecular conformation and inhibits intramolecular rotations. These results are consistent with the higher *Φ*_F_ (0.32) of Y-crystals than G-crystals (*Φ*_F_ = 0.21, Table S1, ESI†). The TPE-EP molecule is characterized by a segregated structure in G- and Y-crystals, which aggregates into layered structures with separated hydrophobic and hydrophilic units. The period distances of the layer structures are 24.6 Å for the Y-crystals (Fig. 2f) and 29.6 Å for the G-crystals (Fig. 2c), respectively.

**Fig. 3:**
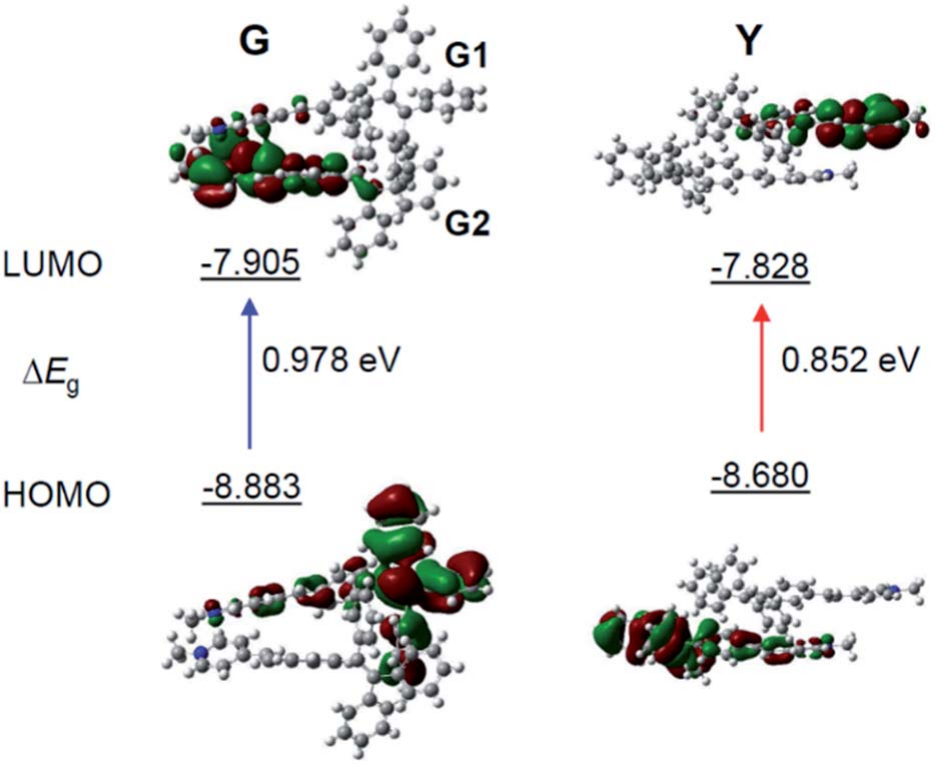
Molecular orbitals of the TPE-EP dimer. The calculated HOMO and LUMO levels for (left) G- and (right) Y-dimers selected from single crystals. Calculations are performed with the B3LYP/6-311G(d,p) basis set using the Gaussian 09 program.

To gain more insight into the mechanism of the fluorescence properties of different polymorphs of G- and Y-crystals, theoretical calculations were carried out using density functional theory (DFT).^42^ The geometry of the monomer and dimer at the ground state was constructed based on the conformers of single crystals. As expected, the HOMO (highest occupied molecular orbital) is mainly contributed by the orbitals of the TPE unit, while the LUMO (lowest unoccupied molecular orbital) is mainly localized on the pyridinium moiety (Fig. 3 and S7, ESI†). Such a D–A structure imparts TPE-EP with a solvatochromic effect (Fig. S8, ESI†).^32^ Unlike the TPE-EP monomers in crystals (Fig. S7, ESI†), the calculated energy gap (Δ*E*_g_) of the Y-dimer (0.852 eV) is smaller than that of the G-dimer (G1/G2, 0.978 eV) (Fig. 3). The change of the energy gap further suggests that intermolecular interactions exert significant effects on the emission red-shift from G- to Y-crystals.^43,44^ Therefore, intervening the crystallization process at the nanometer scale provides an opportunity to control the polymorphism in the condensed state,^15^ notably the molecular packing and interactions between them.

### Polymorph selectivity in polymer phases

**Fig. 4:**
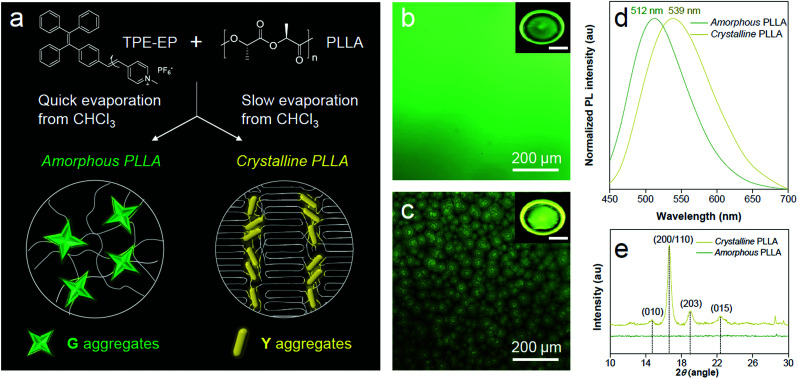
Polymer phase determination by TPE-EP. (a) Preparation of amorphous and crystalline PLLA embedded with TPE-EP. Schematic representation of microenvironment-sensitive fluorescence of TPE-EP in different polymer phases. (b and c) (insets) Fluorescence photos of (b) amorphous and (c) crystalline polymer films and their corresponding magnified images. Excitation wavelength: 365 nm. Inset scale bar: 1 cm. (d) Normalized PL spectra and (e) WAXD patterns of PLLA films embedded with TPE-EP at different phases. The excitation wavelength was 405 nm.

PLLA is a biodegradable and bio-based polymer and is the product resulting from polymerization of l-lactide (Fig. 4a). To impose nano-confinement in PLLA, the polymer crystallization was proceeded to form alternating layers of crystalline lamellae and the amorphous region at the nanometer length scale.^45^ The fluorescence and aggregation of TPE-EP in the PLLA network are shown in Fig. 4a–d. The respective amorphous (Fig. 4b) and crystalline (Fig. 4c) PLLA films were produced by evaporative crystallization, in which polymorphs of TPE-EP were grown within the PLLA network simultaneously. The content of TPE-EP in PLLA was controlled to be 0.1 wt% to simultaneously minimize the influence of TPE-EP on polymer morphology and maintain the bright emission of polymer films. As shown in Fig. 4b and c insets, amorphous and crystalline films show distinct emission under UV irradiation. In the amorphous region, the random molecular jumble lets the chains to cross each other, affording an amorphous polymer network with flexibility and elasticity (Fig. 4a and S9, ESI†). The incorporated TPE-EP molecules in the mobile amorphous region are allowed to orient and pack into aggregates with a structure similar to that of G-crystals (Fig. 4a and b, inset) due to the presence of large free volumes in the amorphous polymer network.^46^ Oppositely, in the crystalline region, the molecular chains are largely locked in place against one another and folded into crystalline lamellae, giving materials strength and rigidity (Fig. 4a and S9, ESI†). Because of the nanoconfined space of the rigid amorphous phase (∼5 nm, Fig. S10, ESI†) between two crystalline layers, the incorporated TPE-EP molecules in Y-aggregations are kinetically trapped in confined space (Fig. 4a and c, inset).^15,17^

When observed under a fluorescence microscope, the homogenous amorphous film shows green emission, while the crystalline film composed of inter-linked spherulites exhibits yellow emission (Fig. 4b and c). The fluorescence properties of the above two films are further confirmed by photoluminescence (PL) spectroscopy (Fig. 4d). It is found that PL spectra of the amorphous PLLA film show an emission maximum at 512 nm, while the crystalline PLLA film exhibits an emission maximum at 539 nm. Both values are very close to those of G- (*λ*_max_ = 507 nm) and Y-crystals (*λ*_max_ = 543 nm). The difference of the emission maximum between bulk single crystals (G- and Y-crystals) and the TPE-EP-embedded polymer matrix (amorphous and crystalline) originates from the imperfect molecular arrangement at the nanoscale. The wide-angle XRD (WAXD) measurements of these two polymer films are performed, further confirming a significant difference in PLLA polymer chain order between the amorphous and crystalline states (Fig. 4e). A broad and structure-less pattern was observed for amorphous PLLA, while the WAXD pattern of the crystalline PLLA shows many sharp peaks that correspond to the α-crystalline phase.^47^ By varying the dye ratio in the polymer matrix from 0.1 to 0.001 wt% and film thickness, analogous experiments were conducted to prepare a series of amorphous and crystalline PLLA films (Fig. S11 and S12, ESI†). The results showed that the emission maximum exerted little change at different TPE-EP concentrations and varied thickness (Fig. S13 and Table S3, ESI†). These data suggest that the aggregation states of guest TPE-EP molecules are mainly dependent on the microenvironment of their host polymer, showing a readily detectable color change to distinguish between amorphous and crystalline PLLA.

The crystalline phase of PLLA comprises stacked lamellae and rigid amorphous regions sandwiched between the lamellae. Embedded TPE-EP molecules are distributed in these amorphous regions as they are driven out of lamellae during crystallization. The lamellar amorphous region has a thickness of ∼5 nm (Fig. S10, ESI†). Moreover, the flexibility of polymer chains in this amorphous region is hindered by the surrounding crystalline region as a polymer molecule changes conformation from the crystalline to amorphous phase.^48^ Consequently, the rigidity of the lamellar amorphous region is higher compared with the mobile amorphous region surrounding the crystalline spherulites. These factors synergistically contribute to the formation and stabilization of Y-crystals in the crystalline phase. Nanoconfined spacing between lamellae can be a favourable factor for the formation of densely packed Y-crystals (unit cell volume is smaller compared to G-crystals). Meanwhile, the rigid environment between lamellae can further stabilize and glassify the metastable Y-crystals.

The incompatibility between amphiphilic TPE-EP and hydrophobic PLLA polymer chains results in formation of the nanocrystalline-like aggregates rather than amorphous aggregates in the polymer matrix (Fig. S14, ESI†). The formation of nanocrystals can be explained by the segregated structure of TPE-EP, which forces the molecules to align in the same direction to take ordered organizations (Fig. S15, ESI†).^49–51^ In addition, the stability of TPE-EP nano-aggregates of G- and Y-crystals in the polymer matrix was also investigated by monitoring the PL spectra for a time period (Fig. S16, ESI†). The stable PL spectra demonstrate the excellent photostability of the fluorescent films at ambient temperature. Moreover, the emission color is unaffected when the film was heated above its glass transition temperature (*T*_g_ = 63 °C, Fig. S17, ESI†). The unaltered emission wavelength suggests the thermal stability of the system.^52,53^

### Crystallinity visualization

**Fig. 5:**
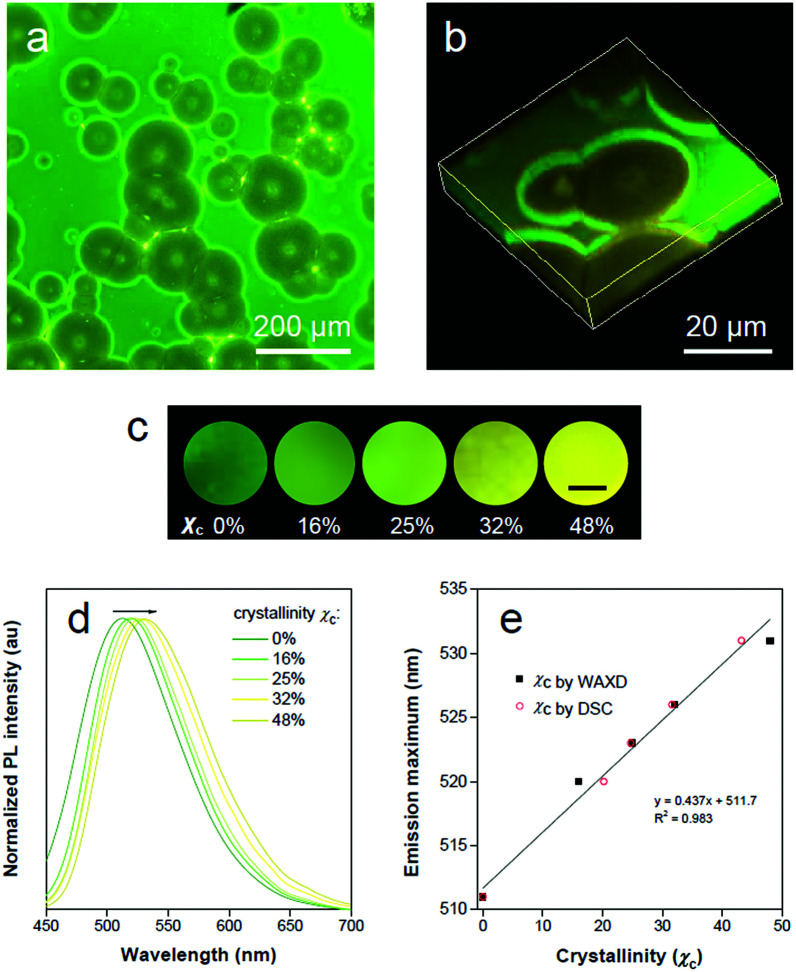
Polymer crystallinity visualization. (a) Overview fluorescence micrograph of the outer surface of the PLLA film with a mixture of crystalline and amorphous regions. (b) Confocal fluorescence 3D images of crystalline spherulites under 405 nm laser irradiation. (c) Fluorescence images of TPE-EP-embedded PLLA at various degrees of crystallinity recorded under 365 nm UV light irradiation. The scale bar is 5 mm. (d) Normalized PL spectra of TPE-EP-embedded PLLA films at various polymer crystallinities. Excitation wavelength: 405 nm. (e) Correlation of the emission maximum with estimated crystallinity with linear-fitting curves.

The highly fluorescent emission contrast of TPE-EP in different phases of PLLA inspires us to explore its potential in detecting microphase distribution and composition *via* polymorph selectivity. Polymer films were obtained *via* controlled evaporative crystallization. The microphase structure of a semi-crystalline polymer is established during materials processing, which is responsible for full understanding of its physical properties. The 2D and 3D microscopic images of the semi-crystalline PLLA films are shown in Fig. 5a and b. Through careful but relatively rapid crystallization from chloroform solution, a film with a mixture of crystalline and amorphous regions was produced (Fig. 5a). Numerous spherulites with circular shapes and yellow emission are apparently recognized and are randomly distributed within the amorphous green region. The distinct fluorescent emission of the polymer crystalline/amorphous phases lights up the whole morphology of the polymer film. The shape and distribution of the spherulites observed were in accordance with the observation under the bright field (Fig. S18, ESI†). The crystalline nature of the polymer spherulites could be supported by a polarized optical microscope (Fig. S19, ESI†). In addition, noticeable boundary rings in bright emission surrounding the crystalline spherulites are clearly observed. Relatively high intensity observed in these boundary rings results from the accumulation of rejected TPE-EP molecules during the crystallization process. The intensity profile of the fluorescence micrograph across amorphous-boundary ring-spherulites indicates the presence of both G- and Y-polymorphs of TPE-EP (Fig. S20, ESI†). The interior morphology can be directly observed through confocal fluorescence microscopy (CFM) based on the different emission properties of G- and Y-aggregates. The confocal image (*XY* plane) was obtained using a linear unmixing method (Fig. S21, ESI†),^54^ showing excellent consistency with the observation under fluorescence microscopy. A total of 22 images in the *XY* plane were collected at different depths using the Z-scan technique, resulting in an architecture of PLLA spherulites in the thin film (Fig. 5b and S22, ESI†). Fluorescence inspection reveals that the noticeable bright rings are interfaces between the amorphous and crystalline polymer regions. Scanning electron microscopy (SEM) was further used to verify the interior morphology (Fig. S23, ESI†). With assistance of solvent (acetone) processing, the amorphous region was etched out to create a pseudo-3D construction.^55^ The SEM image (Fig. S23, ESI†) of the etched film surface shows apparent circular spherulites and a crystalline–amorphous interface, which coincide with those in fluorescence images.

The ratio of G-to-Y nanocrystals of TPE-EP in respective amorphous and crystalline phases could be modified by the degree of polymer crystallinity under different processing conditions to tune the luminescence response of the polymers. Fig. 5c shows the fluorescence response of TPE-EP-embedded PLLA films with crystallinity ranging from 0% to 48%. Crystallinity (*χ*_c_) was estimated by WAXD and differential scanning calorimetry (DSC), respectively (Fig. S24 and Table S4, ESI†). A progressive red-shift from green to yellow was observed, providing a visible crystallinity-detection platform. The fluorescence properties of the above films are further studied by PL spectroscopy. Fig. 5d exhibits the normalized PL spectra of TPE-EP-embedded PLLA films at crystallinities of 0%, 16%, 25%, 32% and 48%, respectively. With an increase in *χ*_c_ (0–48%), the PL spectrum is progressively red-shifted from 511 nm to 531 nm. A linear relationship exists between the emission maximum and *χ*_c_, indicating that polymorphic TPE-EP can serve as a colorimetric marker for crystallinity visualization (Fig. 5e). The crystallinity calculated from DSC and PL data also conform well to a linear relationship. Such a calibration line enables quantitative measurements of *χ*_c_ from the PL change. The results indicate that the polymorph selectivity of the AIE luminogen could not only reveal polymer crystalline phase distribution within the sample, but also provide the average crystallinity over the bulk material. In addition, benefiting from the sensitivity of fluorescence light, our quantitative crystallinity measurements are applicable to the sample with a size even down to micrometers.

### Spatial visualization

**Fig. 6:**
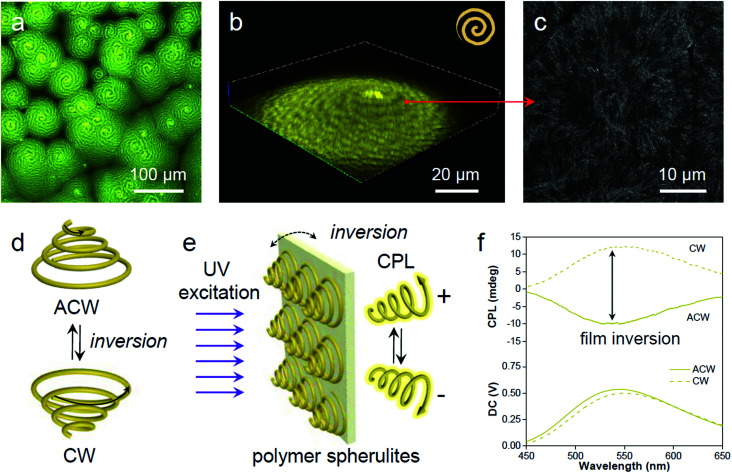
Spatial mapping at the microscopic scale. (a) Representative microscopic fluorescence (2D) and (b) confocal (3D) representation of the PLLA spherulites with alternative bright and dark yellow spirals in the anticlockwise direction. (c) SEM micrograph of a similar spherulite illustrating the interior morphological structure after acetone etching. The red arrow indicates the correlated locations between the fluorescence confocal image and electron micrograph. (d) Enantiomeric chirality switching of the spiral spherulites by film inversion. (e) The spiral spherulites are organized to form a crystalline polymer film, which acts as a chiral medium to generate CPL by preferential reflection of co-handed CPL. (f) Enantiomeric CPL switch upon film inversion.

In the process of evaporative crystallization, because of the imbalanced stress at opposite folding surfaces of PLLA, twisting of the crystalline lamellae occurs (Fig. S25, ESI†).^56^ As a result, banded spherulites with specific handedness are developed because of the radial growth of the helical lamellae from edge-on gradually twisting to flat-on orientations.^57^ A slow evaporation process was then conducted to produce banded PLLA spherulites with long pitch.^58^ The fluorescence microscopic image in Fig. 6a shows the formation of spherulites with alternating bright- and dark-yellow spirals in the anti-clockwise (ACW) direction. Such a microstructure of PLLA spherulites was further verified by confocal analysis (Fig. 6b and S26, ESI†). 3D spherulites with Archimedean spiral organization are presented in the upward vertical direction, which is attributed to the non-diametric sections of 3D spherulites.^59^ The SEM image of the acetone-etched interiors of the PLLA spherulites demonstrates interesting correlations between emission and lamellar assembly (Fig. 6c).^59^ The ridge band in bright-yellow is composed of lamellar bundles, and nano-cracks between the parallel edge-on crystals are visible on the ridge band in SEM images (after solvent etching, Fig. S27, ESI†). Meanwhile, the smooth valley band in dark-yellow is mostly filled by flat-on lamellae.^60^ The bright-yellow emission of the ridge band is mostly ascribed to optical scattering when light penetrates through these fractured and rough places. The ridges and valleys in SEM images conform well to the bright- and dark-yellow spirals observed in a fluorescence and confocal microscope. Fluorescence imaging provides a straightforward and non-invasive method to visualize both the surface and interior of the polymer materials.

The 3D spirals highlighted with yellow emission in polymer spherulites present a good chiroptical activity,^61^ which can divide incident light into left/right-handed circularly polarized light (CPL) components by selective reflection and transmission.^62^ A reversal of the direction provides a clockwise (CW) spiral, resulting in a mirror image of the ACW spiral (Fig. 6d). Upon UV excitation, the AIE-embedded polymer films with chiral structured spherulites are supposed to have an intrinsic ability for CPL generation (Fig. 6e). The film reversal would result in chirality switching while keeping the enantiomeric symmetry. In order to demonstrate the relationship between microscopic morphologies described above and macroscopic optical properties, CPL spectroscopy was conducted to study the chiroptical activity of such polymeric films. The magnitude of CPL can be evaluated using the luminescence dissymmetry factor (*g*_lum_), which equals to 2 (*I*_L_ − *I*_R_)/(*I*_L_ + *I*_R_), where *I*_L_ and *I*_R_ refer to the intensity of left- and right-handed CPL, respectively.^63,64^ In Fig. 6f negative CPL response with an emission maximum at ∼540 nm with a *g*_lum_ of ∼−1.6 × 10^−3^ was observed. Upon flipping the sample, a positive CPL response with a *g*_lum_ of ∼1.6 × 10^−3^ was detected (Fig. S28a, ESI†). The induced CPL spectra show a mirror image upon film inversion, which is modulated by the spiral morphology of the spherulites (Fig. S28b, ESI†). In this regard, the fluorescent AIE luminogen in the polymer matrix simultaneously offers understanding of the spatial organization of the crystalline polymer lamellae and provides a correlation between spiral morphological information and polarized optical signal.

## Conclusions

In summary, we have demonstrated how polymorphic TPE-EP can be selectively grown in semi-crystalline PLLA for constrained space recognition. Nano-confinement provided by the PLLA crystallization process yields metastable polymorph Y while its absence results in thermodynamically stable polymorph G. Consequently, the information on polymer phases (amorphous and crystalline) is marked with distinct color based on the polymorphic states of TPE-EP in specific polymer phases. As a result, the complex hierarchical organization of polymer morphology is transformed into optical signals, which can be seen by color and polarization. We envision that the present concept would be applicable to diverse commodity polymers (Fig. S29, ESI†) through a proper molecular structure design. These morphological sensing properties have potential for an *in situ* monitoring polymer manufacturing process, in turn, to predict the materials' physical properties. Furthermore, the synergic effects between luminescent molecular assemblies and polymer micro-structures account for versatile luminescence manipulation, including intensity, color, and polarization, providing continuous emissive materials in developing foldable devices and wearable systems.

## Conflicts of interest

There are no conflicts to declare.

## Supplementary Material

SC-011-C9SC04239C-s001

SC-011-C9SC04239C-s002
