# Supplementary material for: Polymorph selectivity of an AIE luminogen under nano-confinement to visualize polymer microstructures
Source: Chem Sci. 2019 Nov 22;11(4):997–1005. doi: 10.1039/c9sc04239c (PMC8146380; doi:10.1039/c9sc04239c)
Supplement: SC-011-C9SC04239C-s001 [file SC-011-C9SC04239C-s001.pdf]

Electronic Supplementary Material (ESI) for Chemical Science.
This journal is © The Royal Society of Chemistry 2019

Supporting Information

Polymorph Selectivity of AIE Luminogen under Nano-Confinement to Visualize Polymer Microstructure

Michidmaa Khorloo, Yanhua Cheng,* Haoke Zhang, Ming Chen, Herman H. Y. Sung, Ian D. Williams, Jacky W. Y. Lam and Ben Zhong Tang*

This PDF file includes:

Tables **S1** to **S5**

Figures **S1** to **S29**

Experimental Section

Materials. TPE-EP was synthesized according to the synthetic methods reported previously.[1] All starting materials and reagents were purchased from commercial suppliers and used as received. The PLLA (M_w = 8.9 × 10^4 $gmol^{-1}$, PDI = 1.2) was purified by dissolving in chloroform and precipitated in methanol and was dried in a vacuum oven before use.

Single crystal and X-ray analysis. All crystals in this work were obtained at room temperature. **G**-crystals were cultivated through vapor diffusion of dichloromethane/hexane mixtures of TPE. **Y**-crystals were prepared through slow evaporation of tetrahydrofuran (THF)/hexane mixtures. **O**-crystals were obtained through slow evaporation of THF mixtures. Measurement of the single crystals were collected on a SuperNova Dual Atlas diffractometer using graphite monochromated Cu K_α radiation (λ = 1.54184 Å). The diffraction data were collected at 100 K and the crystallographic parameters are summarized in Table S2. The structure was solved with the ShelXS structure solution program using direct methods in Olex2[2] and refined with ShelXL

refinement package using Least Squares minimization. Non-hydrogen atoms were refined anisotropically while hydrogen atoms were refined using the riding model.

Preparation of TPE-EP embedded PLLA film. TPE-EP embedded PLLA film preparation procedures are as follows: TPE-EP was dissolved in THF to prepare a 1 mg mL^{-1} stock solution. 50 µL of the above TPE-EP solution was mixed with 50 mg of PLLA in chloroform (10 mg mL^{-1}) under stirring. Unless specified otherwise, the content of TPE-EP in PLLA matrix was controlled to be 0.1 wt%. Afterward, the mixed solution was put in a covered weighing bottle with a radius and height of 2.5 cm and 3.0 cm, respectively. Crystallization of PLLA was occurred by controlling the solvent evaporation rate at ambient temperature. Then, the thin PLLA film was peeled off from the glass bottle for further study.

CFM imaging. CFM measurements were carried out on a Zeiss LSM7 DUO confocal microscope. For TPE-EP-embedded PLLA film with a mixture of amorphous and crystalline phases, CFM images were first obtained through two-channel scanning under laser excitation of 405 nm. Channel one was selected between 410-516 nm and channel two was selected between 518-688 nm. The obtained images were linearly unmixed[3] using ZEN software into two components, each of which was respectively assigned by green and yellow as shown in Figure S21 and S22. For TPE-EP-embedded crystalline PLLA film, CFM images were obtained under laser excitation of 405 nm, and the optical signal was selected between 400-700 nm (Figure S26).

Measurements. Molecular weight and polydispersity index (PDI) of purified PLLA was determined by Waters Association Gel Permeation Chromatography (GPC) system equipped with UV detectors. Differential Scanning Calorimetry (DSC) measurements were conducted using a TA DSC Q1000 under nitrogen flow at a heating rate of 10 ^{o}C min^{-1}. Photoluminescence

(PL) spectra were measured on a Horiba Fluorolog-3 spectrofluorometer. Quantum yields of the solid samples and polymer films were determined on a Horiba Quanta using a calibrated integrating sphere. Fluorescence lifetime measurements were conducted on a FLS 980 spectrometer. Fluorescent and optical microscopy images were captured on a Nikon Eclipse 80i microscope. WAXD analysis was conducted on a X'pert PRO, PANanalytical diffractometer using Cu-K radiation. Small-angle X-ray scattering (SAXS) experiments were performed on a SAXSess instrument (Anton Paar). Nanoindentation tests were conducted using a Hysitron TI950 TriboIndenter. SEM images were obtained on a JEOL-7100F SEM. Normal photographs were taken on Canon 7D camera under 365 nm UV illumination. CPL spectra were carried out on JASCO CPL-300, the measurement conditions of CPL are listed in Table S5.

Crystallinity measurements. Percentage crystallinities (χ_c) of the polymer films were estimated by DSC and XRD methods. For DSC measurements, crystallinities of the polymer films were calculated from DSC thermal profiles according to equation (1).

$$\chi_c\,(\%) = \frac{\Delta H_m - \Delta H_c}{\Delta H_m^0} \times 100\% \qquad (1)$$

ΔH_m is melting enthalpy and ΔH_c is exothermal enthalpy arising from crystallization in the polymer film during heating process. ΔH^0_m is the melting enthalpy of PLLA crystal, 106 J g^{-1}.[4] DSC thermographs of tested films are shown in Figure S24b and thermal profiles indicating enthalpies and temperatures of melting and crystallization of the films are shown in Table S4. For XRD measurements (Figure S24a), crystallinities of the polymer films were calculated by dividing the area under the crystalline peaks to overall area of the diffractometer after subtraction of the background signal. Strong peak at 2θ of 16.7° from (200)/(110) planes and relatively smaller peak at 2θ of 19.0° from (203) planes were selected as crystalline peaks for fitting.

Lamellae thickness calculation. Lamellae thickness and long period were calculated from SAXS patterns using one-dimensional (1D) correlation function.[5] The normalized 1D correlation function was extracted from Lorentz-corrected experimental SAXS results by a Fourier transform by equation (2).

$$\gamma_x = \frac{\int_0^\infty I(q)q^2 \cos(qx)dq}{\int_0^\infty I(q)q^2 dq} \quad (2)$$

The long period L is the first maximum value of first oscillation, and the thickness of the thinner phase (L_t, determined as amorphous phase) is the intercept between level of the first minimum and the extrapolation of linear part of the correlation function as labelled in Figure S10.

Single crystal data. CCDC #1917740 for **G**-crystals and 1917741 for **Y**-crystals contains the supplementary crystallographic data for this paper. These data can be obtained free of charge from The Cambridge Crystallographic Data Centre via www.ccdc.cam.ac.uk/data_request/cif.

Results and Discussion

Table S1. The photophysical properties of polymorphic TPE-EP. Emission maximum wavelength (λ_{em}), fluorescence quantum yield (Φ_F), average lifetime (τ), radiative rate constant (k_r) and non-radiative rate constant (k_{nr}).

Compound	Fluorescence λ_{em} (nm)	Φ_F [a)]	τ (ns) [b)]	k_r [c)] (×10^8 s^{-1})	k_{nr} [c)] (×10^8 s^{-1})
TPE-EP-**G**	507	0.21	2.1	1.0	3.8
TPE-EP-**Y**	543	0.32	2.3	1.4	2.9
TPE-EP-**O**	575	0.23	2.4	1.0	3.2

a) Φ_F measured by an integrating sphere.

b) Fluorescence lifetimes (τ) calculated according to equation of $\tau = \frac{\sum_i a_i \tau_i^2}{\sum_i a_i \tau_i}$, τ_i is an individual lifetime and a_i is an amplitude of multi-exponential decay fit.

[c] The radiative rate constant $k_r = \Phi_F/\tau$; the non-radiative rate constant $k_{nr} = 1/\tau - k_r$.

Table S2. Crystal data and structure refinement for G- and Y-crystals.

Crystal	TPE-EP-**G**	TPE-EP-**Y**
Empirical formula	$C_{69}H_{58}Cl_2F_{12}N_2P_2$	$C_{34}H_{28}F_6NP$
Formula weight	1276.01	595.54
Temperature, K	100.03(11)	100.01(10)
Crystal system	monoclinic	monoclinic
Space group	$P2_1/n$	$P2_1/c$
a, Å	11.2525(2)	24.8939(7)
b, Å	9.21940(13)	9.1783(2)
c, Å	59.1397(9)	12.5794(3)
α, °	90	90
β, °	93.8053(14)	98.168(3)
γ, °	90	90
Volume, Å^3	6121.68(17)	2845.01(13)
Z	4	4
ρ_{calc}, g/cm^3	1.384	1.390
μ, mm^{-1}	2.143	1.422
F(000)	2632.0	1232.0
Crystal size, mm^3	0.2 × 0.02 × 0.02	0.2 × 0.08 × 0.02
Radiation	CuKα (λ = 1.54184)	CuKα (λ = 1.54184)
2θ range for data collection, °	7.918 to 134.996	10.772 to 134.998
Index ranges	$-13 \leq h \leq 13$, $-11 \leq k \leq 10$, $-35 \leq l \leq 70$	$-29 \leq h \leq 28$, $-10 \leq k \leq 10$, $-15 \leq l \leq 13$
Reflections collected	32777	14173
Independent reflections	10976 [R_{int} = 0.0416, R_{sigma} = 0.0423]	4687 [R_{int} = 0.0309, R_{sigma} = 0.0300]
Data/restraints/parameters	10976/86/849	4687/6/389
Goodness-of-fit on F^2	1.036	1.019
Final R indexes [$I>=2\sigma (I)$]	R_1 = 0.0623, wR_2 = 0.1501	R_1 = 0.0869, wR_2 = 0.2128
Final R indexes [all data]	R_1 = 0.0744, wR_2 = 0.1577	R_1 = 0.0932, wR_2 = 0.2163
Largest diff. peak/hole, e. Å^{-3}	0.95/-0.71	0.95/-0.43

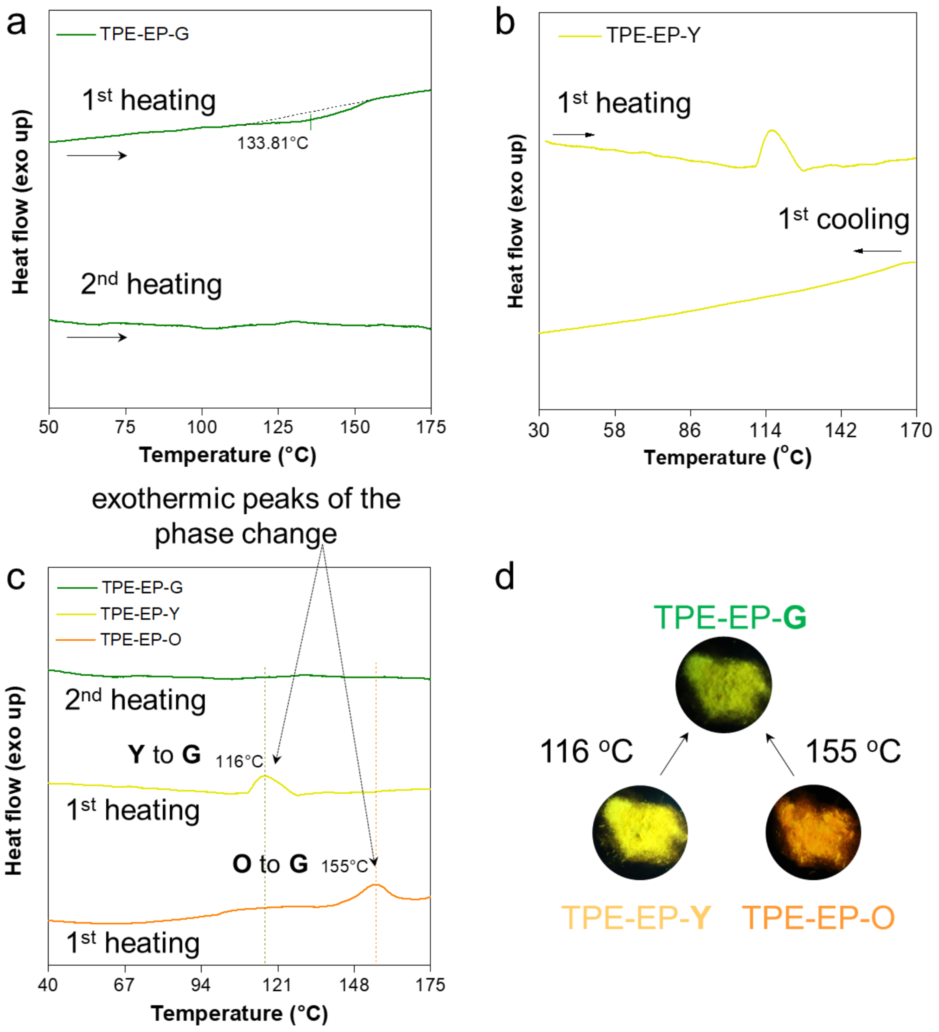

Figure S1. DSC analysis of TPE-EP polymorphs and their fluorescent photographs. (**a**) DSC traces of **G**-crystals at first (upper) and second heating (bottom) processes. (**b**) DSC traces of **Y**-crystals at first heating (upper) and cooling (bottom) processes. (**c**) DSC traces of **G**-, **Y**- and **O**-crystals with the phase-transition temperatures. (**d**) Heat-triggered phase transformation. The luminescent color changes of **Y** to **G**, and **O** to **G** were initiated at 116 °C and 155 °C, respectively.

In Figure S1a, the first heating scan of **G**-crystals revealed a broad endothermic peak at ~134 °C, indicating the evaporation of included solvent in crystalline lattice. The disappearance of the broad peak at the second heat scan further demonstrates the exclusion of the solvent molecules. In Figure S1b, a thermal phase transformation from **Y** to **G** was initiated at 116 °C.

No reverse phase was observed when the sample was cooled. In Figure S1c, a similar thermal phase transformation from **O** to **G** was observed at 155 °C. The DSC analyses indicate that **Y**-crystals and **O**-crystals are metastable while **G**-crystals are thermodynamically stable. The luminescent color changes of TPE-EP polymorphs were shown in Figure S1d.

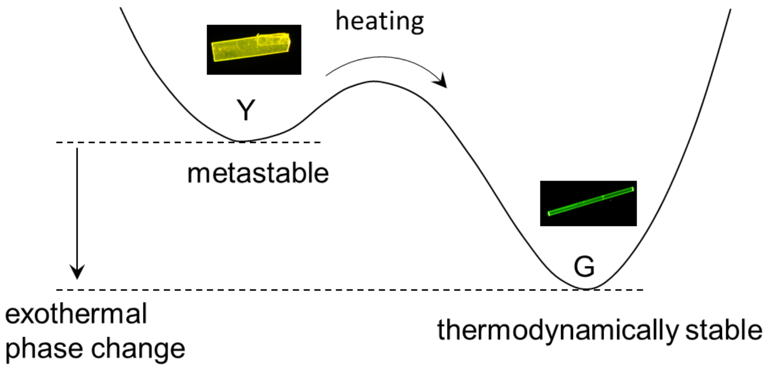

Figure S2. Diagram of the thermodynamic energies for **G**- and **Y**-crystals.

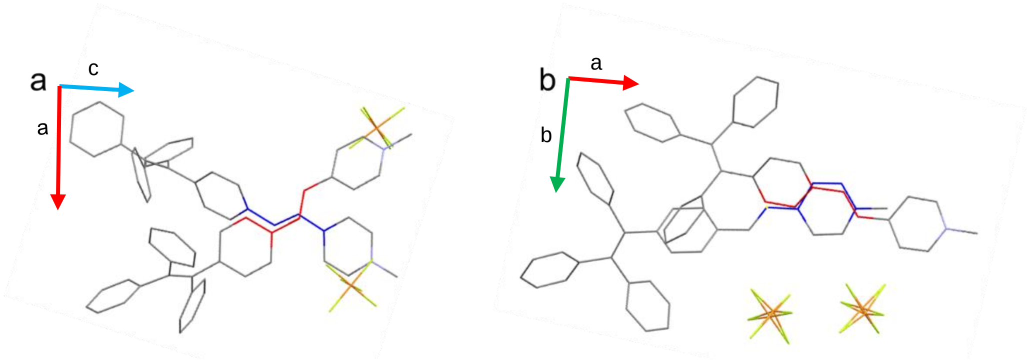

Figure S3. Molecular overlap of the (**a**) **G**-crystals and (**b**) **Y**-crystals.

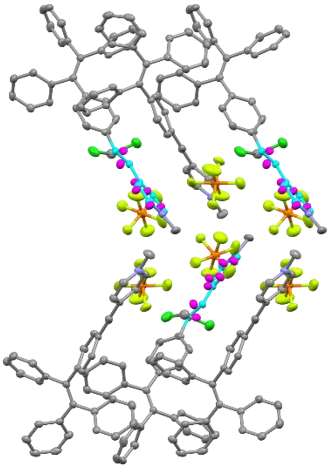

Figure S4. Ellipsoid drawing of the crystal structure of **G** with crystallographically ordered units (**G**1) and crystallographically disordered units (**G**2) with two conformations. Conformers I and II are colored with blue and pink, respectively. The hydrogen atoms in **G**-crystals are omitted for clarity.

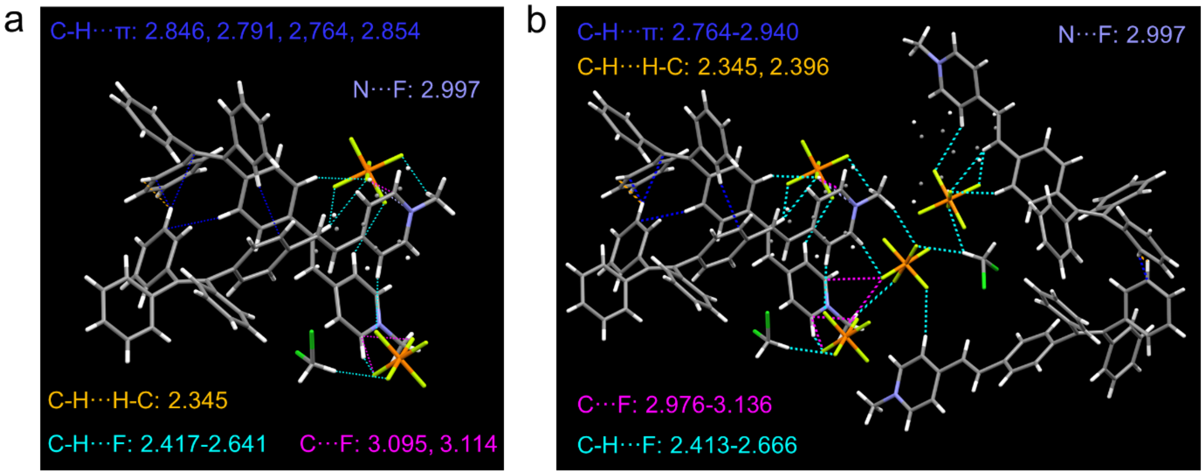

Figure S5. Multiple interactions in G-crystals. (**a**) Multiple intermolecular interactions between **G**1 and **G**2. (**b**) Intermolecular packing viewed along the *b* axis.

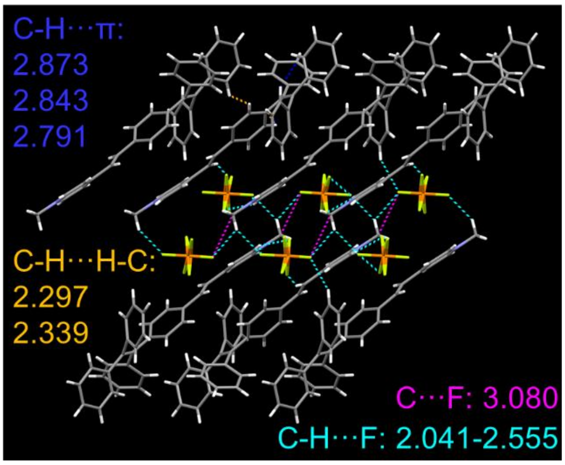

Figure S6. Multiple interactions in Y-crystals. Short contacts in **Y**-crystals and intermolecular packing viewed along the *b* axis.

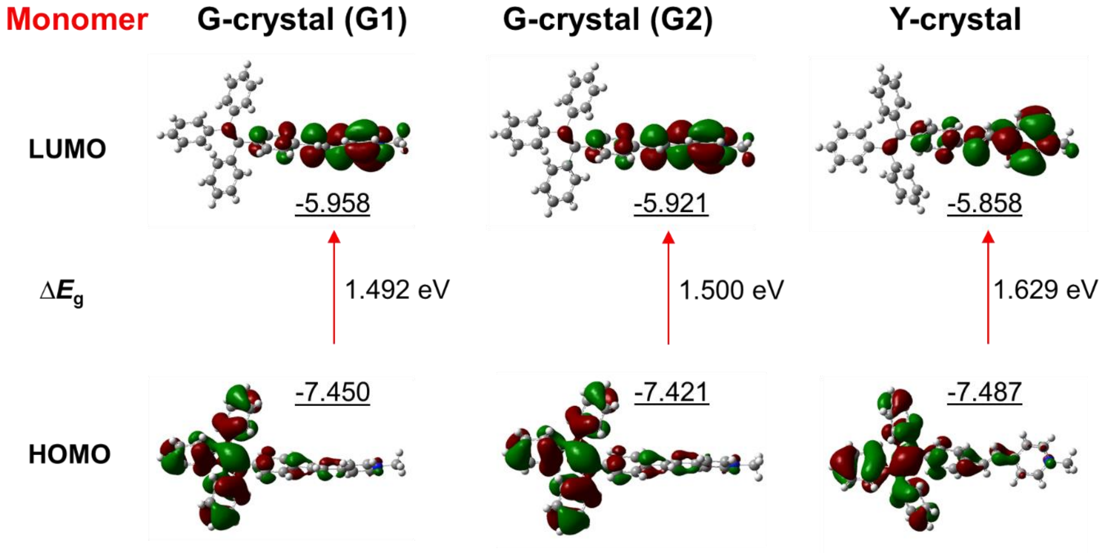

Figure S7. Calculated HOMO and LUMO levels for TPE-EP monomer selected from single crystals. The calculation is obtained from B3LYP/6-311G(d,p) basis set by Gaussian 09 program.

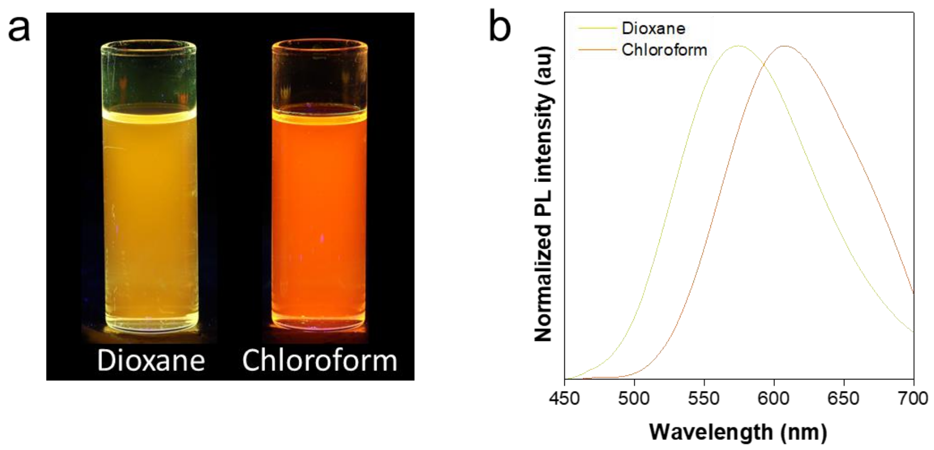

Figure S8. Solvatochromic properties of TPE-EP. (**a**) Photographs of the solutions of TPE-EP in organic solvents of dioxane and chloroform taken under UV light. Excitation wavelength: 365 nm. (**b**) Normalized fluorescence spectra of TPE-EP in dioxane (yellow line) and chloroform (orange line).

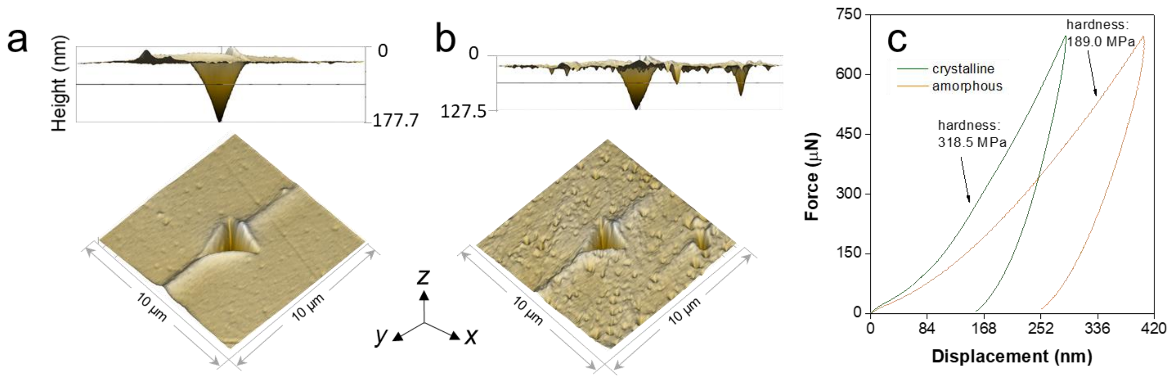

Figure S9. Mechanical properties of amorphous and crystalline PLLA studied by nanoindentation test. Nanoindentation images of (**a**) amorphous and (**b**) crystalline PLLA films. (**c**) Force-displacement curves of the amorphous (green line) and crystalline (yellow line) PLLA films.

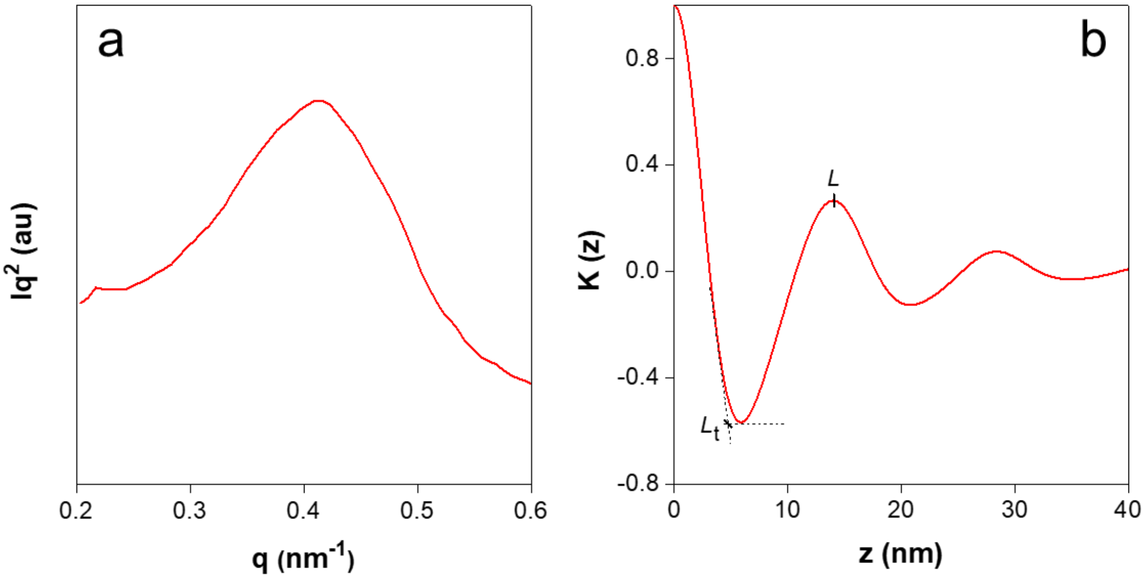

Figure S10. (**a**) Lorentz-corrected SAXS pattern of TPE-EP-embedded crystalline PLLA and (**b**) corresponding 1D correlation function profile. The long period (L = 14 nm) of crystalline PLLA was determined by the position of first peak, while the average thickness of the thinner layers (L_t = 5 nm) was calculated by the connection between tangent and baseline of the first wave trough.[6]

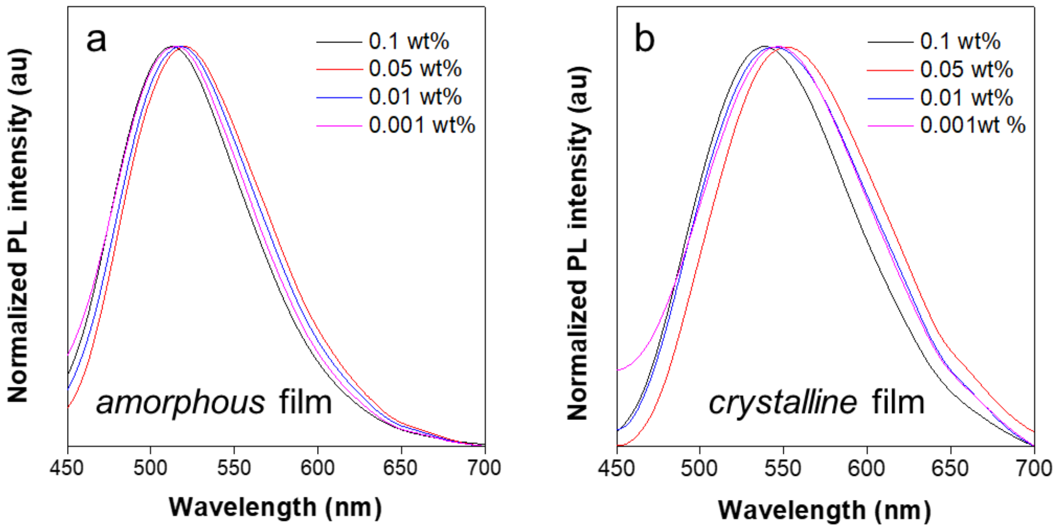

Figure S11. Normalized PL spectra of TPE-EP-embedded (**a**) amorphous and (**b**) crystalline PLLA films with various doping ratio.

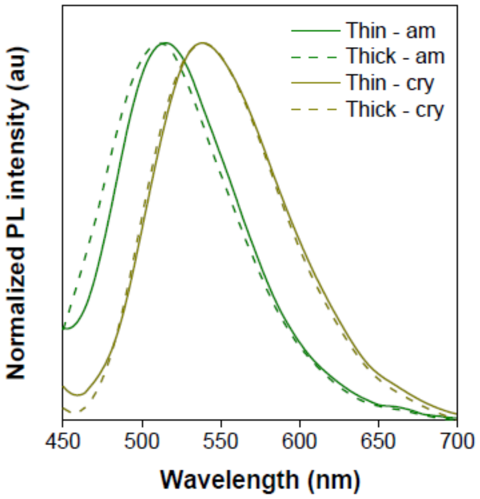

Figure S12. PL spectra of amorphous and crystalline films with different thickness.

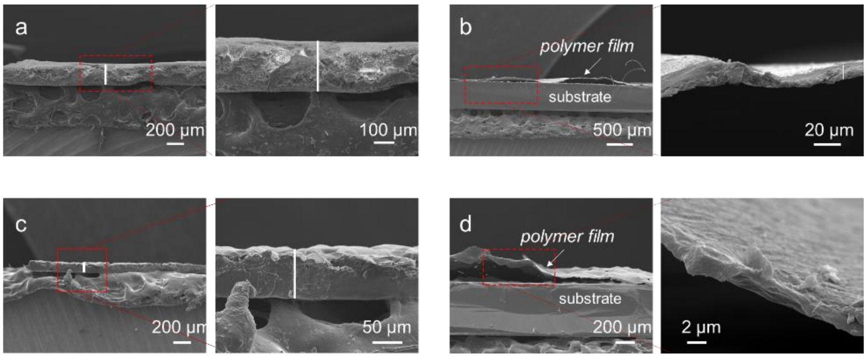

Figure S13. Cross-section SEM images of (**a**, **c**) thick and (**b**, **d**) thin amorphous (top) and crystalline (bottom) film.

Table S3. The film thickness and emission property of TPE-EP-embedded PLLA film.

Samples	Thickness (μm)	Emission maximum (nm)
Thin-am	10	511
Thick-am	200	511
Thin-cry	1.7	539
Thick-cry	82	539

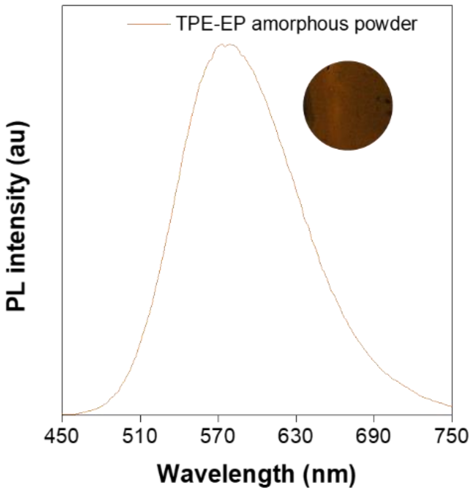

Figure S14. The PL spectrum of TPE-EP amorphous powder. Inset: image of amorphous TPE-EP powder under 365 nm UV light. The amorphous powder of TPE-EP shows orange emission.

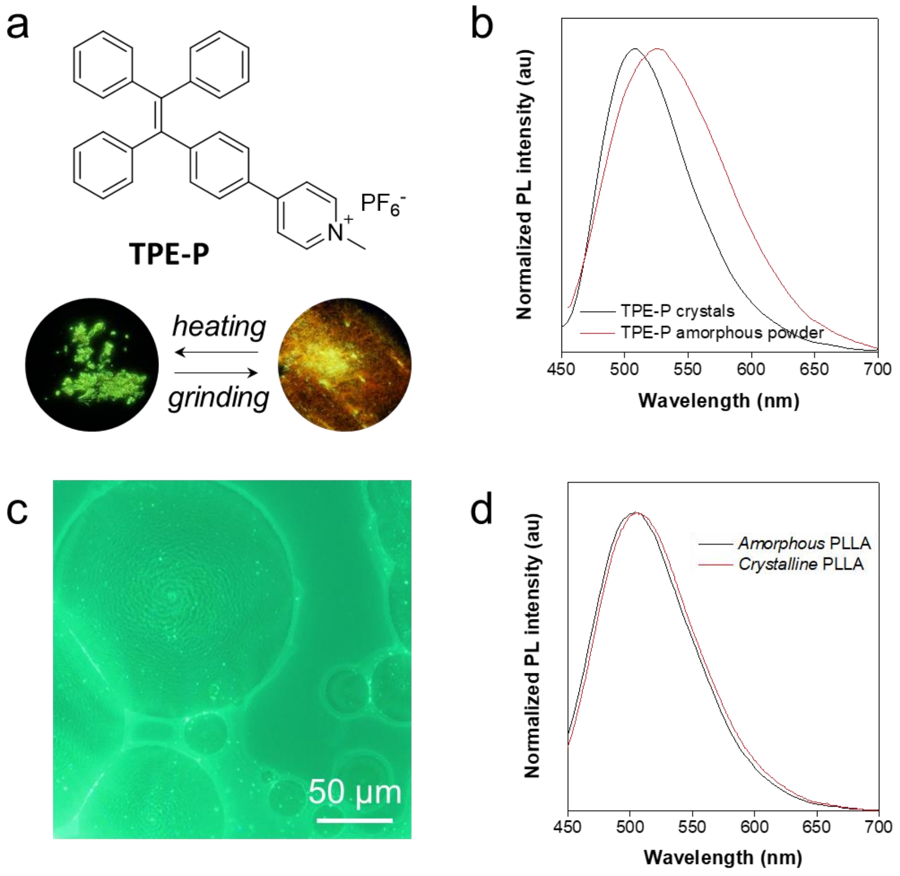

Figure S15. Control demonstration by TPE-P. (**a**) (top) Chemical structure and (bottom) fluorescent images of TPE-P taken under 365 nm UV light. Bottom left: green emissive crystal,

bottom right: orange amorphous powder. (**b**) Respective PL spectra of TPE-P crystals and TPE-P amorphous powder. (**c**) Fluorescence micrograph of the TPE-P-embedded PLLA film with a mixture of crystalline and amorphous regions. (**d**) Normalized PL spectra of TPE-P-embedded PLLA films at different phases.

Control experiment with TPE-P. In order to further prove the nanocrystalline states of TPE-EP in PLLA, we use another AIE molecule showing similar chemical structure with that of TPE-EP, namely TPE-P. In TPE-P, the pyridinium moiety is directly attached to TPE unit (Figure S15a). For TPE-P molecules, only crystalline crystals and amorphous powders are observed, which show green (λ_{em} = 508 nm) and yellow (λ_{em} = 526 nm) emission, respectively (Figure S15b). Experiments were performed to fabricate polymer films that embedded with TPE-P, similar to that for TPE-EP-embedded PLLA films. In Figure S15c and d, both amorphous and crystalline PLLA phases that embedded with TPE-P show green emission (λ_{em} = ~506 nm). The emission maximum is very close to that of TPE-P crystals. The results further demonstrate that segregated AIE molecules are in forms of nanocrystals rather than amorphous aggregates in PLLA.

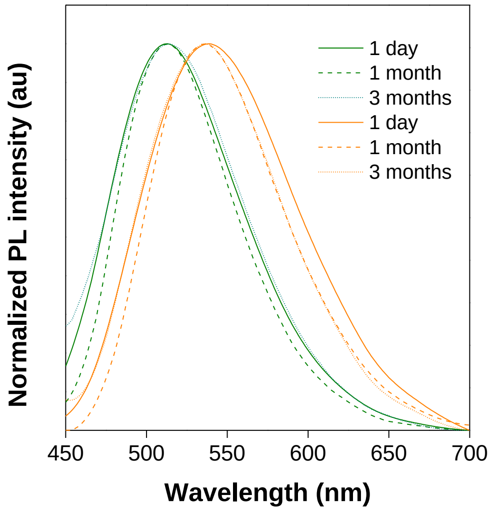

Figure S16. PL spectra of TPE-EP-embedded PLLA films monitored during three months. Green lines: amorphous PLLA films embedded with TPE-EP; Orange lines: crystalline PLLA films embedded with TPE-EP.

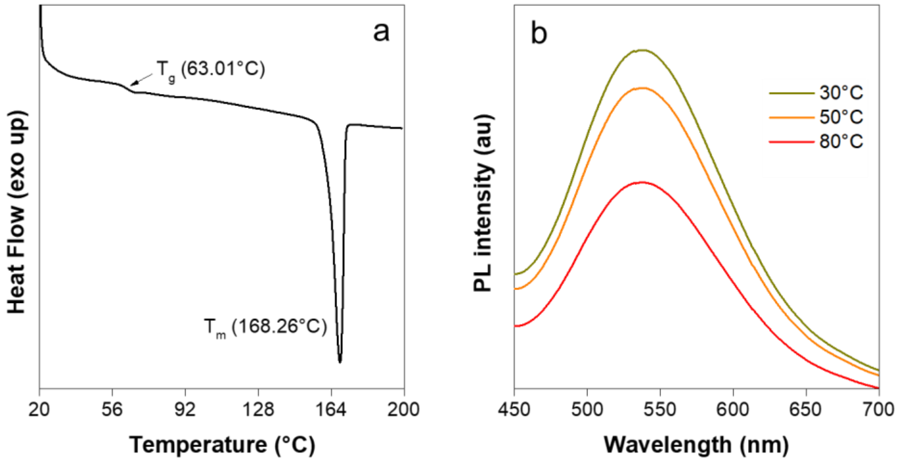

Figure S17. (**a**) DSC analysis of PLLA with glass transition temperature and melting temperature. (**b**) PL spectra of TPE-EP embedded PLLA film at different temperatures.

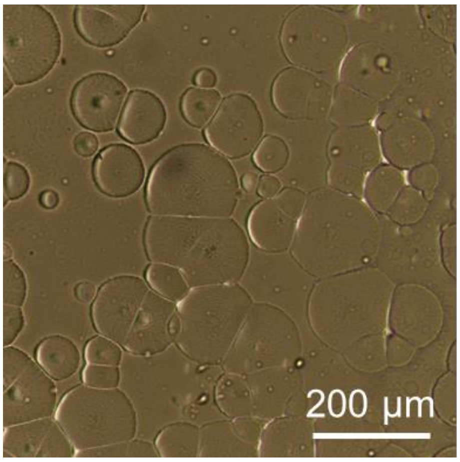

Figure S18. Microscopic image of the PLLA films taken under bright filed.

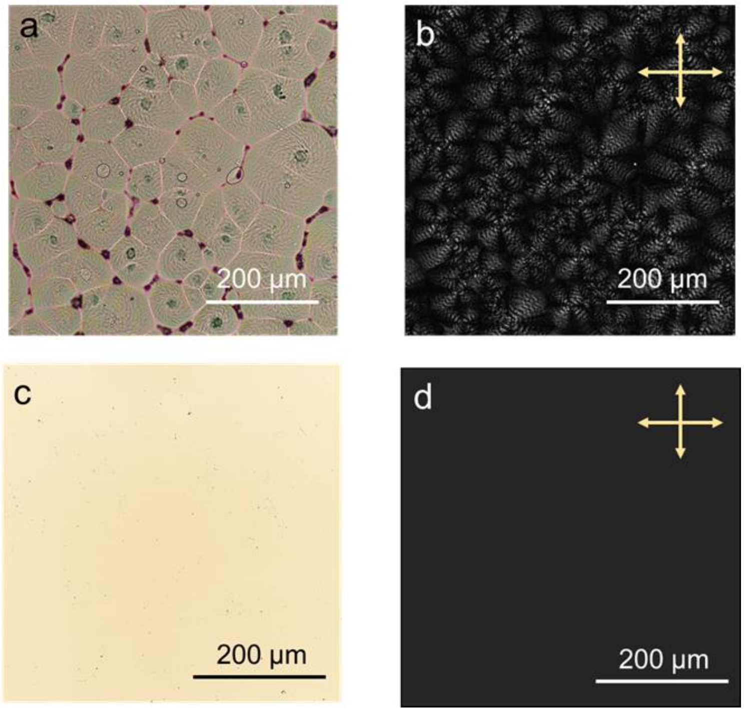

Figure S19. (**a**, **c**) Bright field and (**b**, **d**) polarized optical micrograph of crystalline (top) and amorphous (bottom) films.

Polarized optical microscopy (POM) was used to image the crystalline structure. Crystalline films (top) with spherulites show birefringence with the Maltese cross and extinction bands in accordance with the bright field image. In contrast, POM of amorphous film (bottom) between two crossed polarizers shows no birefringence.

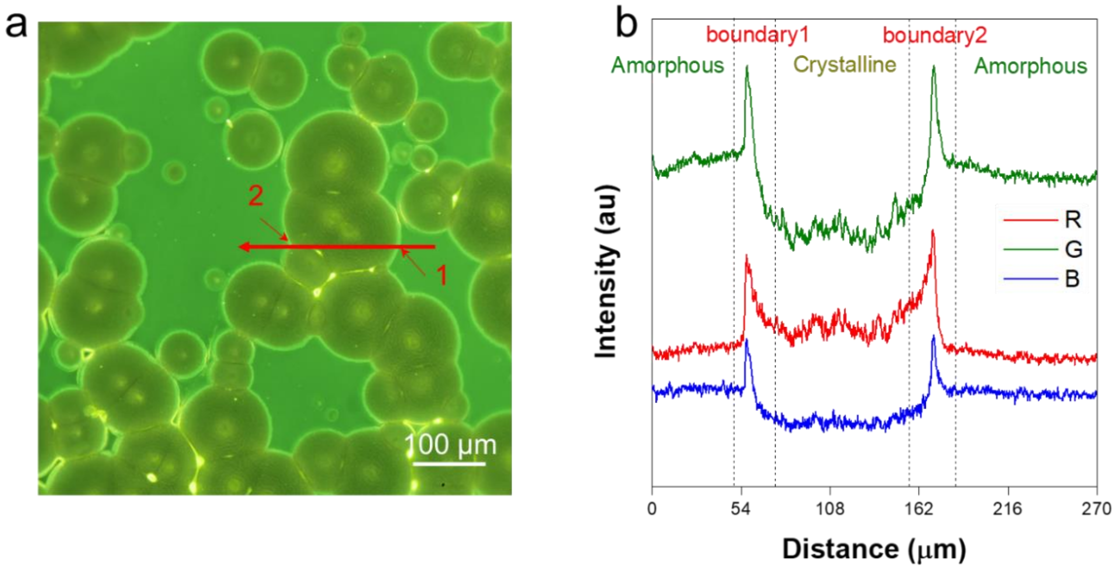

Figure S20. (**a**) Fluorescent micrograph with the arrow indicating the route for intensity profile. (**b**) Intensity profiles of RGB component along the arrow shown in **a**.

Bright rings bounding the spherulites comprise of both amorphous and crystalline arrangement based on the SEM data in Figure S23. The intermixed nature of the phase interface consequently causes TPE-EP to form mixture of green and yellow emissive state. These bright rings show emission intensity higher than amorphous and crystalline regions. The underlying reason for the observation is relatively higher amount of TPE-EP in this interface region due to accumulation of rejected TPE-EP molecules around edges of the spherulites.[7] Intensity profiles of red, blue, and green component of the fluorescent micrograph (along the red arrow) in Figure S20 confirms that the edge area shows highest intensity in all three components. R, G, and B percentage of the intensity profile in the boundary ring is 29%, 53%, and 18%, respectively. This set of value lies between the values of amorphous (25%, 59%, 16%) and crystalline (34%, 54%, 12%) region in terms of red and blue component, further validating that TPE-EP in the boundary rings exist as a mixture of TPE-EP with yellow and green emission.

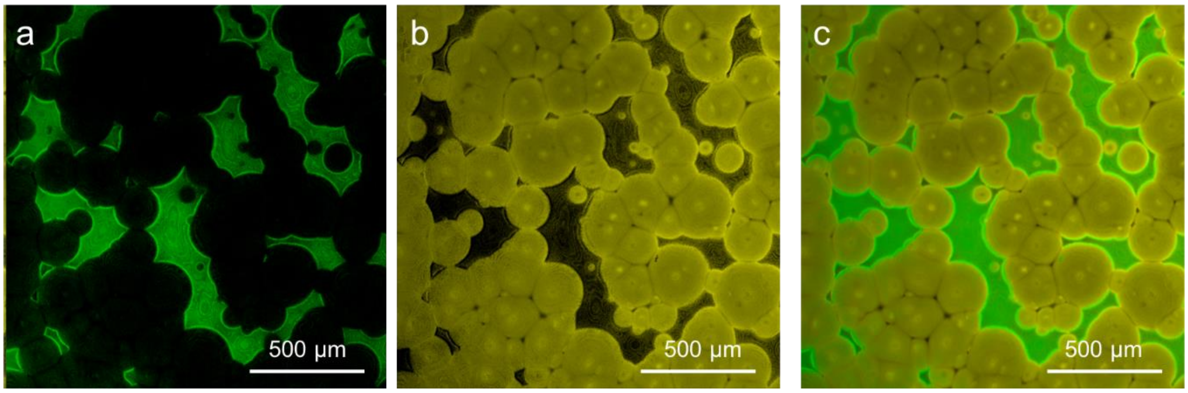

Figure S21. Linearly unmixed CFM images of TPE-EP-embedded PLLA with a mixture of crystalline and amorphous regions. (**a**) Amorphous PLLA doped by TPE-EP-**G** shown as green and (**b**) crystalline PLLA doped by TPE-EP-**Y** shown as yellow in confocal image by linear unmixing two-channel imaging. Channel one of green was selected between 410-516 nm and Channel two of yellow was selected between 518-688 nm. (**c**) Merged images of panels **a** and **b**.

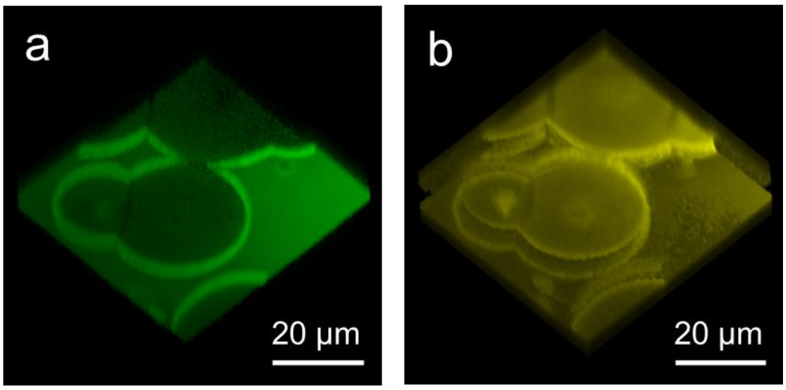

Figure S22. Confocal fluorescent 3D images of crystalline spherulites under 405 nm laser irradiation. (**a**) Amorphous PLLA doped by TPE-EP-**G** shown as green and (**b**) crystalline PLLA doped by TPE-EP-**Y** shown as yellow in confocal image using linear unmixing two-channel Z-scanning. Channel one was selected between 410-516 nm and Channel two was selected between 518-688 nm.

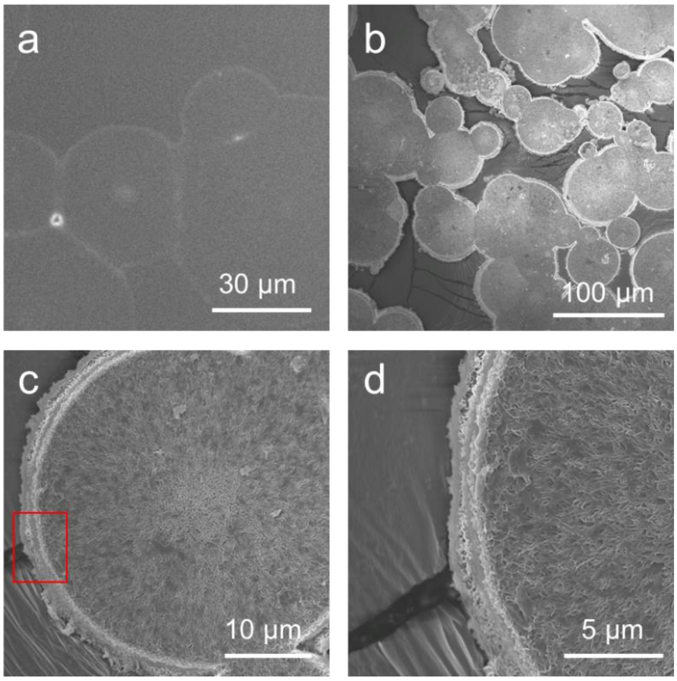

Figure S23. SEM image of PLLA film with a mixture of amorphous and crystalline regions (**a**) before and (**b**, **c, d**) after acetone etching. (**c**) SEM image of the crystalline spherulites and (**d**) magnified image of the selected area in **c**.

After gold sputtering, the morphology of the film is presented as shown in Figure S23a. In contrast, only ambiguous polymer crystalline shape can be observed in the SEM image. It has been reported that the density of amorphous PLLA (1.248 g ml^{-1}) is less than that of crystalline states (1.290 g ml^{-1}).[8] Acetone could dissolve the amorphous PLLA rather than the crystalline region. As a result, SEM images of 3D spherulites shown in Figure S23b, c and d were created.

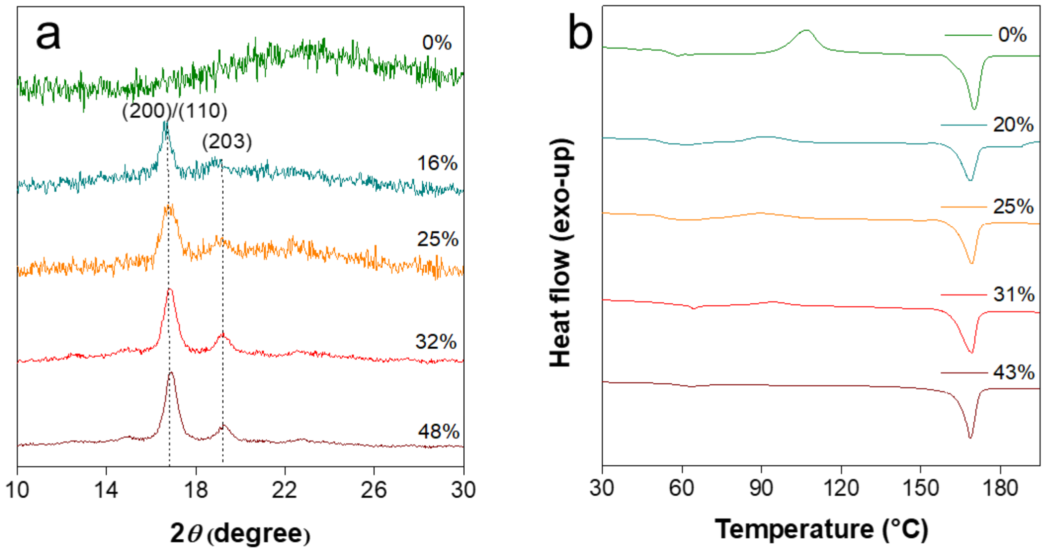

Figure S24. Crystallinity measurement by WAXD and DSC. (**a**) WAXD profiles of TPE-EP-embedded PLLA at different polymer crystallinity. (**b**) DSC thermograms of TPE-EP-embedded PLLA at different crystallinity, which were recorded under nitrogen at the first heating scan at a rate of 10 °C min^{-1}. Corresponding melting enthalpy values, exothermal enthalpy values, crystallization temperatures, melting temperatures, and crystallinity values are listed in Table S4.

Table S4. Thermal profiles of PLLA with varied crystallinity.

T_c (°C)	ΔH_c (J g^{-1})	T_m (°C)	ΔH_m (J g^{-1})	χ_c (%)
107.0	-37.52	170.1	37.28	**0**
92.02	-12.65	162.8	34.05	**20.2**
89.46	-10.37	163.3	36.52	**24.7**
94.14	-6.551	161.4	39.58	**31.6**
/	/	165.1	45.80	**43.2**

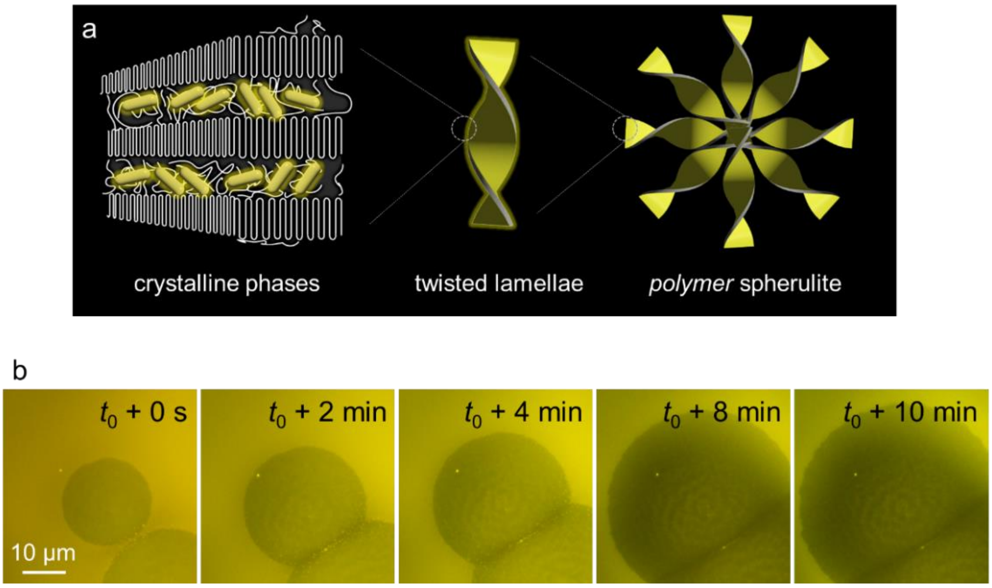

Figure S25. (**a**) Illustration of the formation of spiral-banded spherulite that doped with TPE-EP.[6] TPE-EP molecules in forms of nanocrystalline **Y** are distributed in between two PLLA lamellas. (**b**) Real-time fluorescent photos of the evolution process of one period (10 min) in a PLLA spherulite.

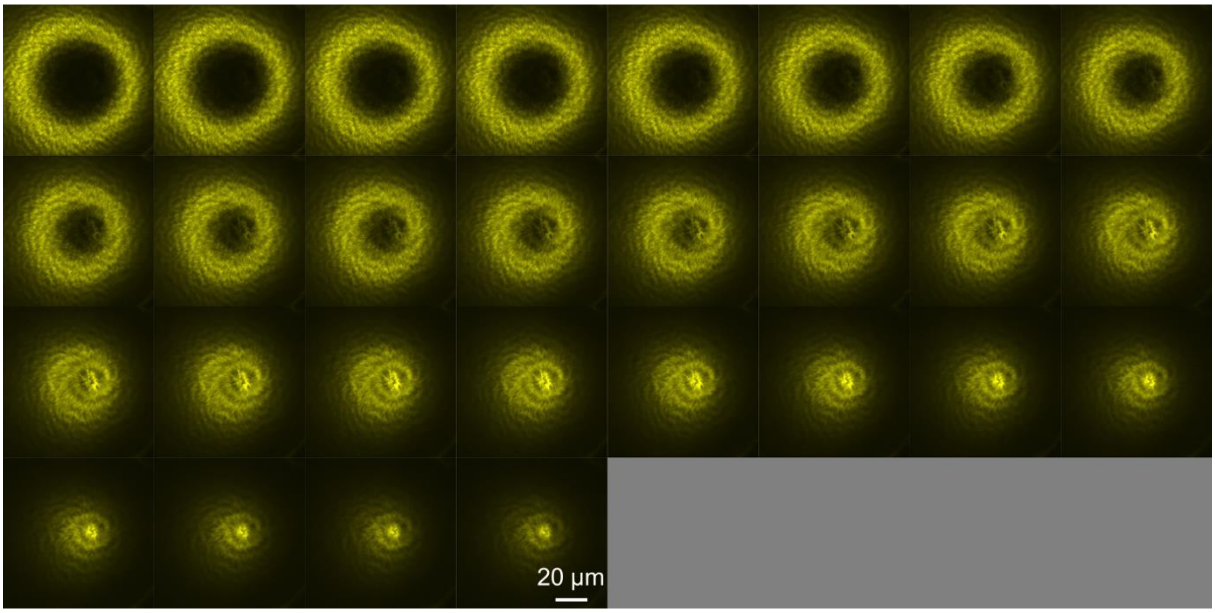

Figure S26. Confocal images of each slice of spiral banded spherulites embedded with TPE-EP using Z-scan technique. Excitation wavelength: 405 nm. The optical signal was selected between 400-700 nm. The images clearly show the Archimedean spiral structure of PLLA

spherulites. The 3D spiral highlighted with bright-yellow is presented in the upward vertical direction.

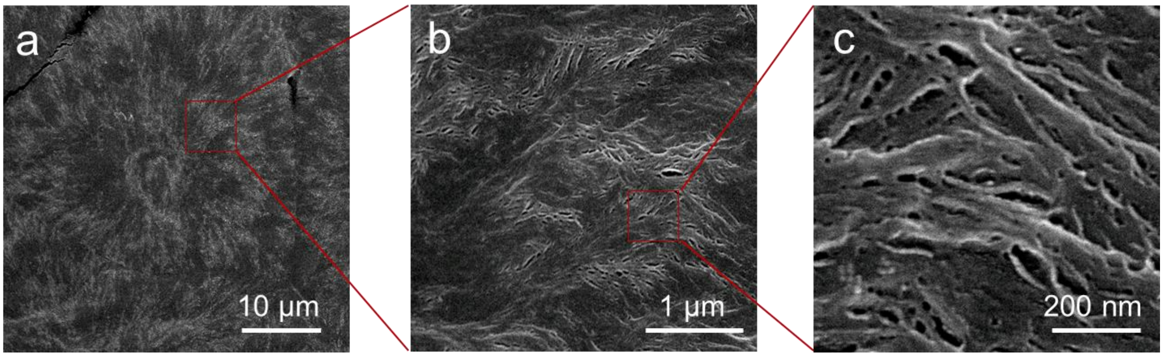

Figure S27. SEM images of (**a**) solvent etched crystalline spherulite and (**b, c**) magnified images of the respective marked area.

Table S5. The measurement conditions of CPL measurements on CPL-300 instrument.

**CPL Measurement Conditions**	
Excitation Wavelength	340 nm
Excitation Slit Width	3 mm
Data Pitch	0.1 nm
Measure Range	660 - 360 nm
Emission Slit Width	3 mm
Scan Speed	50 nm/min
D.I.T.	8 sec

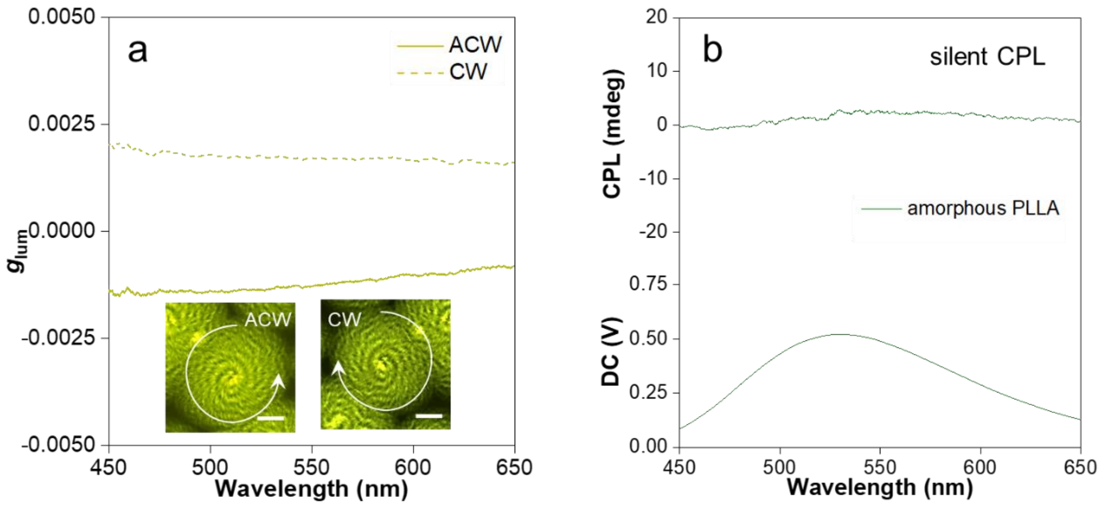

Figure S28. (**a**) CPL dissymmetry factor g_{lum} as a function of the wavelength. The spectra were recorded on the TPE-EP-embedded crystalline film that excited from two different sides. Solid line: Excited from the side that the spiral turn direction was anti-clockwise (ACW). Dash line: Excited from the side that the spiral turn direction was clockwise (CW). The spiral morphology of polymer spherulites observed from different sides are shown in the insets. Scale bars are 20 μm. (**b**) CPL spectrum of amorphous PLLA film that embedded with TPE-EP, which show silent signal. The results indicate that the CPL response is originated from the superstructure of the spiral patterns.

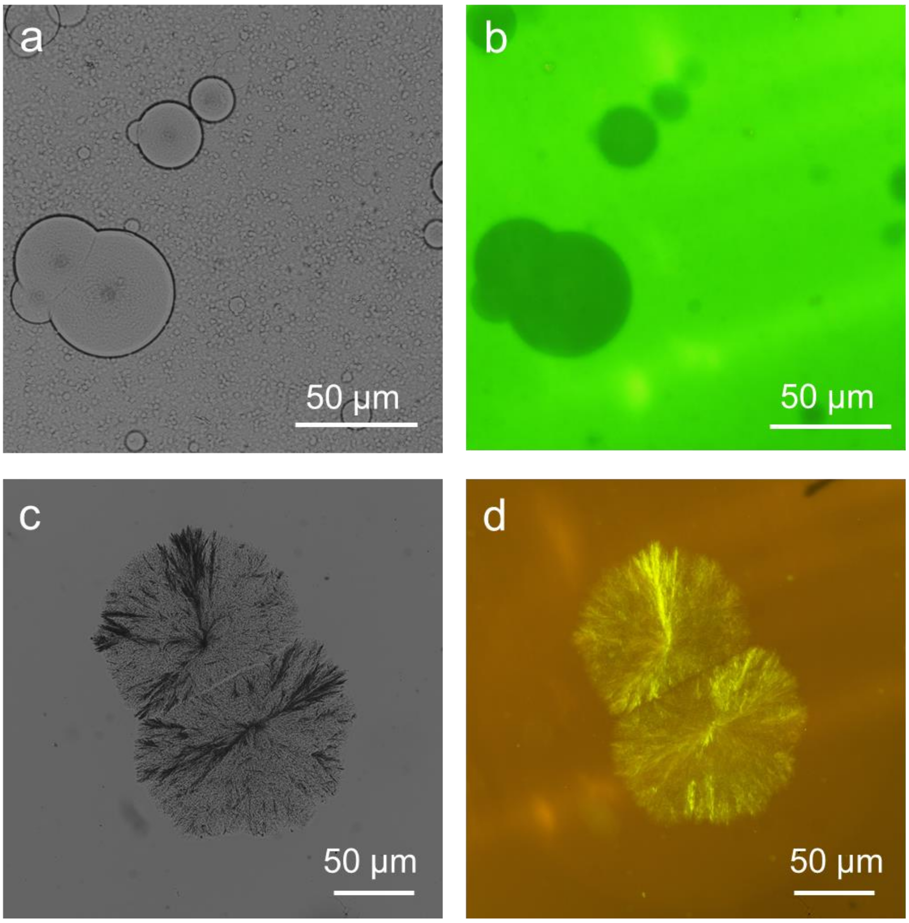

Figure S29. (**a**, **c**) Bright field and (**b**, **d**) fluorescent micrograph of TPE-EP in PHBV (top) and PEG (bottom) polymer, respectively.

The sensitivity of TPE-EP to distinguish polymer phases in other semi-crystalline polymers were studied. Melt crystallization method was used to obtain the polymer film with a mixture of crystalline and amorphous regions. The doping ratio of TPE-EP in PHBV and PEG was controlled to be 0.1 wt%. For PHBV (top), it is found the crystalline region shows dark emission, while amorphous region exhibits bright green emission. The distinct "On" and "Off" emission in respective amorphous and crystalline phase indicates the responsiveness of TPE-EP in polymer phases. In crystalline PHBV, TPE-EP molecules were excluded out from the spherulites due to the densely packed polymer lamellae, leading to significantly decreased intensity in the crystalline spherulites. We propose that TPE-EP molecules also form nano-

aggregates with a similar structure of **G**-crystals in amorphous PHBV, which is attributed to the similar chemical structure of PHBV and PLLA.

For PEG (bottom), it found the crystalline region shows yellow emission, while amorphous exhibits orange emission. The result also shows potential of TPE-EP to distinguish the polymer phases of PEG. In contrast to the mechanism of TPE-EP in PLLA, the distinct emission color in amorphous and crystalline regions is originated from twisted intramolecular charge-transfer effect of the TPE-EP molecular rotors. In amorphous PEG, the hydrophilic network provides a polar microenvironment for TPE-EP to emit orange emission. We speculate the yellow emission of TPE-EP in crystalline PEG results from the confined space of crystalline lamellae and comparatively low polarity of crystalline PEG network.

References

[1] N. Zhao, M. Li, Y. Yan, J. W. Y. Lam, Y. L. Zhang, Y. S. Zhao, K. S. Wong, B. Z. Tang, *J. Mater. Chem. C* **2013**, *1*, 4640-4646.

[2] O. V. Dolomanov, L. J. Bourhis, R. J. Gildea, J. A. K. Howard, H. Puschmann, *J. Appl. Cryst.* **2009**, *42*, 339-341.

[3] T. Zimmermann, J. Rietdorf, R. Pepperkok, *FEBS Lett.* **2003**, *546*, 87-92.

[4] J.-R. Sarasua, R. E. Prud'homme, M. Wisniewski, A. Le Borgne, N. Spassky, *Macromolecules* **1998**, *31*, 3895-3905.

[5] F. Auriemma, G. C. Alfonso, C. De Rosa, *Polymer Crystallization II: From Chain Microstructure to Processing, Springer International Publishing*, **2016**; pp 127-165.

[6] M.-C. Li, H.-F. Wang, C.-H. Chiang, Y.-D. Lee, R.-M. Ho, *Angew. Chem. Int. Ed*. **2014**, *53*, 4450-4455.

[7] H. Keith and F. Padden Jr, *J. Appl. Phys.* 1964, **35**, 1270-1285.

[8] K. M. Nampoothiri, N. R. Nair, R. P. John, *Bioresour. Technol.* **2010**, *101*, 8493-8501.
